# Reactive microglia partially envelop viable neurons in prion diseases

**DOI:** 10.1172/JCI181169

**Published:** 2024-10-03

**Authors:** Natallia Makarava, Tarek Safadi, Olga Bocharova, Olga Mychko, Narayan P. Pandit, Kara Molesworth, Simone Baiardi, Li Zhang, Piero Parchi, Ilia V. Baskakov

**Affiliations:** 1 Center for Biomedical Engineering and Technology and; 2 Department of Neurobiology, University of Maryland School of Medicine, Baltimore, Maryland, USA.; 3 University Department of Biomedical and Neuromotor Sciences (DiBiNeM), University of Bologna, Bologna, Italy.; 4 IRCCS, Istituto delle Scienze Neurologiche di Bologna, Programma Neuropatologia delle Malattie Neurodegenerative, Bologna, Italy.; 5 Department of Physiology, Center for Vascular and Inflammatory Diseases, University of Maryland School of Medicine, Baltimore, Maryland, USA.

**Keywords:** Infectious disease, Neuroscience, Innate immunity, Neurodegeneration, Prions

## Abstract

Microglia are recognized as the main cells in the central nervous system responsible for phagocytosis. The current study demonstrates that in prion disease, microglia effectively phagocytose prions or PrP^Sc^ during early preclinical stages. However, a critical shift occurred in microglial activity during the late preclinical stage, transitioning from PrP^Sc^ uptake to establishing extensive neuron-microglia body-to-body cell contacts. This change was followed by a rapid accumulation of PrP^Sc^ in the brain. Microglia that enveloped neurons exhibited hypertrophic, cathepsin D–positive lysosomal compartments. However, most neurons undergoing envelopment were only partially encircled by microglia. Despite up to 40% of cortical neurons being partially enveloped at clinical stages, only a small percentage of envelopment proceeded to full engulfment. Partially enveloped neurons lacked apoptotic markers, but showed signs of functional decline. Neuronal envelopment was independent of the CD11b pathway, previously associated with phagocytosis of newborn neurons during neurodevelopment. This phenomenon of partial envelopment was consistently observed across multiple prion-affected brain regions, various mouse-adapted strains, and different subtypes of sporadic Creutzfeldt-Jakob disease (sCJD) in humans. The current work describes a phenomenon of partial envelopment of neurons by reactive microglia in the context of an actual neurodegenerative disease, not a disease model.

## Abstract 2

Our study showed that in prion diseases, microglia change their mission with disease progression, form clearance of harmful prions to enveloping alive neurons.

## Graphical abstract

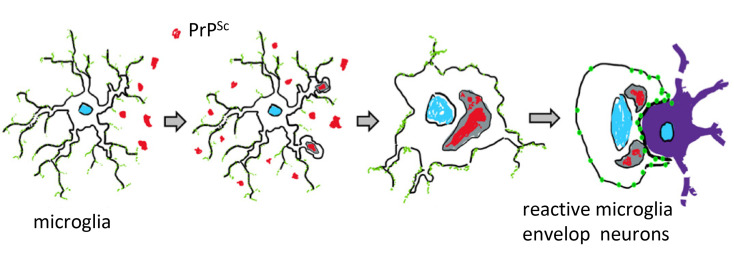

## Introduction

Microglia are recognized as the main cells in the CNS responsible for phagocytosis. In early brain development, microglia are crucial in optimizing neural circuitry and eliminating excessive synapses, neurons, astrocytes, and oligodendrocytes (1, 2). Throughout adult life, microglia defend the CNS against invading pathogens and clean up tissues from apoptotic cells and myelin debris. During normal aging and neurodegenerative diseases, microglia encounter new challenges, as they are responsible for clearing protein aggregates and cell debris, while simultaneously participating in repair and regeneration processes. As a response to ongoing changes in brain homeostasis and persistent exposure to protein aggregates, microglia acquire various reactive phenotypes, some of which are characterized by upregulated phagocytic pathways (3–6). It has been proposed that the upregulation of the phagocytosis under conditions of chronic neuroinflammation leads to phagocytosis of viable neurons by microglia, contributing to neurodegeneration (7). Indeed, it appears that the phagocytic pathways responsible for pruning of synapses and neurons during neurodevelopment also contribute to synaptotoxicity in neurodegenerative diseases and aging (7–10). However, most of the evidence that microglia can phagocytose viable neurons was collected in vitro using cultured cells, whereas detecting engulfment of viable neurons in actual neurodegenerative disease has been difficult (11–13).

Phagocytosis comprises several sequential steps, encompassing the recognition, engulfment, and degradation of a target (14). When microglia encounter a target, they extend filopodia, forming a phagocytic cup that engulfs the target and gives rise to phagosomes (14). Subsequently, phagosomes undergo maturation by merging with endosomes and lysosomes, forming phagolysosomes, where the phagocytosed material is digested. In vivo, the entire process of microglia-mediated phagocytosis of an individual cell is completed in less than 2 hours (15).

Prion diseases, also known as transmissible spongiform encephalopathies, represent a group of transmissible neurodegenerative disorders affecting both humans and animals (16). These conditions currently lack any effective treatment, and their invariably fatal outcome is well established. Prion diseases are instigated by prions or PrP^Sc^, which is the misfolded, aggregated form of a cellular sialoglycoprotein known as the prion protein or PrP^C^ (17). The pathogenic mechanism involves the replication and dissemination of prions throughout the CNS, achieved by recruiting and converting host-expressed PrP^C^ molecules into misfolded, β-sheet–rich PrP^Sc^ states (18). In individuals or animals infected with prions, the neurodegeneration is attributed to PrP^Sc^ accumulation, which exerts a toxic effect on neurons (19–25).

Over the years, the idea that microglia constitute the primary host defense against prions has gained strong experimental support (26–28). Indeed, the ablation of microglia, either before prion infection or during the initial stages of the disease, accelerated disease progression (29–32). Furthermore, the knockout of MFGE8, a factor secreted by microglia that mediates the phagocytosis of apoptotic bodies, resulted in a 40-day acceleration of prion pathogenesis and elevated levels of PrP^Sc^ (33). Contrary to the notion that microglia are protective, partial inhibition of microglia proliferation and reactivity at the preclinical stage (98 to 126 days after infection with the ME7 strain) delayed the onset of behavioral signs and extended survival by 26 days (34). Moreover, the inhibition of microglia activation through the administration of an immunosuppressant just before or at the time of disease onset suppressed reactive gliosis and prolonged the survival of humanized mice infected with human prions (35). In response to prion infection, microglia were shown to upregulate IFN-1, which activates phagolysosomal pathways (36). Surprisingly, the knockout of the *IFNAR1* gene, which encodes a receptor for IFN-1, ameliorated clinical signs, improved neuronal survival along with synaptic density, and prolonged survival in ME7-infected mice (36).

This study reveals that microglia initially help to keep PrP^Sc^ levels low by efficiently phagocytosing the protein during the early preclinical stage of prion disease. However, as the disease progresses, microglia shift their activity from PrP^Sc^ uptake to forming close contacts with neurons, resembling partial engulfment of neuronal cells. This change in behavior precedes the rapid accumulation of PrP^Sc^ and is associated with hypertrophic lysosomal compartments in microglia. Surprisingly, only a small fraction of neurons are fully engulfed, and partially enveloped neurons, while not apoptotic, show signs of functional decline. This partial neuronal envelopment is consistently observed across different prion-affected brain regions, mouse strains, and human sporadic Creutzfeldt-Jakob disease (sCJD) subtypes.

## Results

### Reactive microglia extend into the pyramidal layer of the CA1 region of the hippocampus.

Among known mouse-adapted prion strains, in C57BL/6J mice, SSLOW caused disease with the shortest incubation time (mean ± SD 123 ± 4 days post inoculation [dpi] to terminal disease via intracerebral inoculation [i.c.] route). In the SSLOW-infected C57BL/6J host, PrP^Sc^ accumulated in multiple brain regions, including the cortex, thalamus, caudate putamen, and hippocampus (Supplemental Figure 1; supplemental material available online with this article; https://doi.org/10.1172/JCI181169DS1). These same brain regions developed spongiform vacuolation and profound neuroinflammation, as seen by immunostaining for reactive microglia and astrocytes (Supplemental Figure 1 and Supplemental Figure 2, A and B).

**Figure 1:**
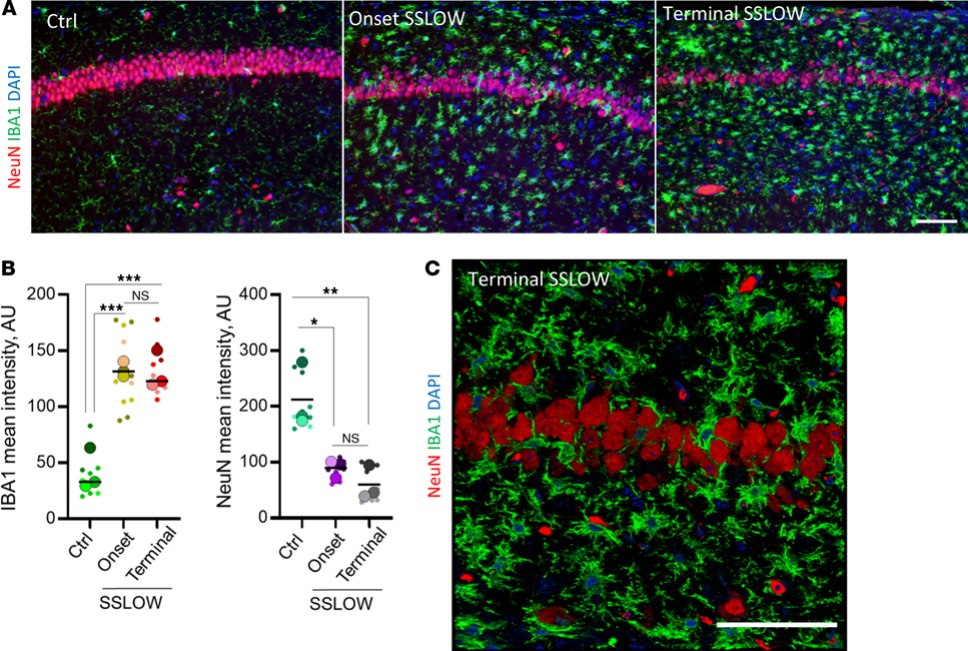
Reactive microglia in prion-infected mice extend into pyramidal layer of CA1 area of hippocampus. The CA1 area of hippocampus in noninfected age-matched control C57BL/6J mice (Ctrl) and C57BL/6J mice infected with SSLOW via i.c. examined at clinical onset and terminal stage using staining with anti-IBA1 (a marker of microglia, **A** and **C**) and anti-NeuN (a nuclear neuronal marker, **A** and **C**). (**B**) Quantification of the mean intensity of IBA1 and NeuN signals in pyramidal layer of hippocampus. Colors represent different brains. Dots represent mean intensity values in individual fields of view. Average values for each brain are shown as circles. Black lines mark means. *n* = 3 animals per group. *P < 0.05; **P < 0.01; ***P < 0.001, ordinary 1-way ANOVA followed by Tukey’s multiple-comparison tests. (**C**) 3D reconstruction of confocal microscopy images of reactive microglia invading pyramidal layer in terminal SSLOW-infected mice. Scale bars: 50 μm (**A**); 100 μm (**C**).

By the clinical onset of the disease, marked thinning of the pyramidal layer in the CA1 area of hippocampus was observed in SSLOW-infected C57BL/6J mice (Figure 1, A and B). Neuronal loss in the pyramidal layer coincided with an increase in density of reactive microglia (Figure 1, A–C). The microglia extended processes around neuronal somas or intruded as whole cells into the neuronal layer (Figure 1, B and C). Extensive microglia-neuronal contacts in the CA1 area prompted us to examine other brain regions affected by prions.

### In SSLOW-infected mice, reactive microglia envelop neurons.

**Figure 2:**
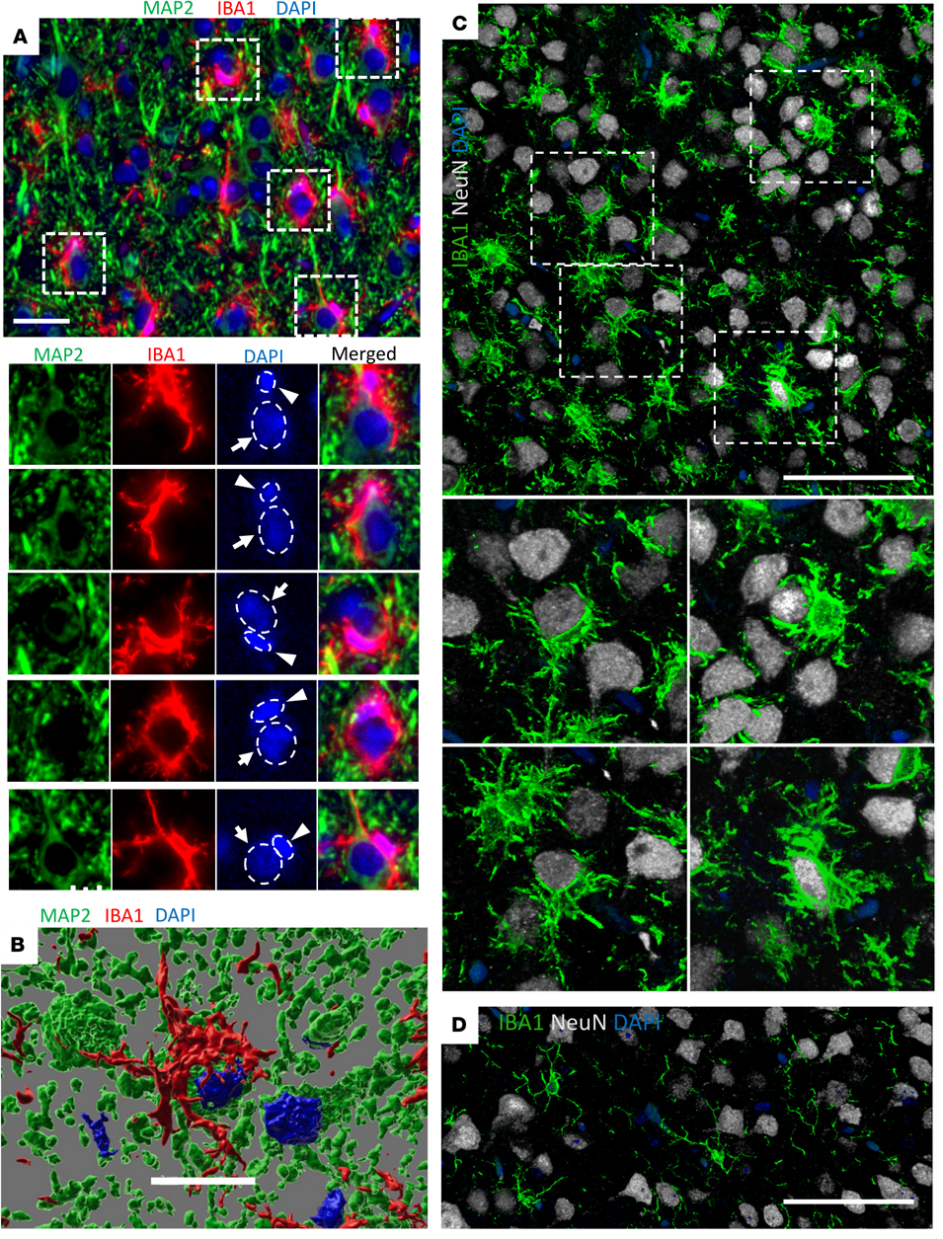
Reactive microglia in cortexes of prion-infected mice envelop neuronal soma. Terminally ill C57BL/6J mice infected with SSLOW via i.p. (**A** and **B**) or i.c. routes (**C**) or noninfected age-matched controls (**D**), stained with anti-IBA1 (**A**–**D**) and anti-MAP2 (**A** and **B**) or anti-NeuN (**C** and **D**) antibodies and examined by epifluorescence microscopy (**A**) or confocal microscopy following by 3D reconstruction (**B**–**D**). Smaller panels in **A** and **B** show enlarged images of individual microglial cells that partially or fully envelopes neuronal soma. In **A**, dashed circles mark nuclei, arrows and arrowheads point at neuronal and microglial nuclei, respectively. Scale bars: 20 μm (**A**); 10 μm (**B**); 50 μm (**C** and **D**).

Coimmunostaining of clinically ill C57BL/6J mice infected with SSLOW via intraperitoneal (i.p., 159 ± 13 dpi to terminal disease, mean ± SD) or intracranial (i.c., 123 ± 4 dpi to terminal disease) routes revealed a substantial population of cortical neurons partially or fully enveloped by the soma of reactive microglia (Figure 2, A–C, and Supplemental Figure 3, A–D). In noninfected cortices, such contacts between neurons and microglial soma were very rare or absent (Figure 2D and Supplemental Figure 3A). Three-dimensional reconstruction of confocal imaging further illustrates partial neuronal envelopment by the soma of reactive microglia in prion-infected mice (Figure 2B and Supplemental Figure 4, A and B, Supplemental Video 1, and Supplemental Video 2).

Microglia typically execute phagocytosis by forming pseudopodia with a phagocytic cup (14, 37, 38). This cup engulfs the target, which may include synapses, apoptotic cells, or protein aggregates. Full engulfment of a target by the phagocytic cup results in the formation of phagosomes within pseudopodia. Consistent with this mechanism, pseudopodia with phagocytic cups were observed in adult 5XFAD mice, where microglia partially engulfed Aβ aggregates or neurons (Supplemental Figure 3, E and F). In contrast to the traditional phagocytic mechanism involving pseudopodia and a phagocytic cup, in prion-infected mice, neurons were surrounded by microglial soma (Figure 2, A–C, and Supplemental Figure 3, B–D). The proximity of microglial and neuronal nuclei supports the notion that neurons were encircled by microglial somas, which often exhibited a characteristic cup-shaped appearance (Figure 2A and Supplemental Figure 3D) rather than by extended pseudopodia. Envelopment of individual neurons by microglia in prion-infected brains occurred at a one-to-one ratio (Figure 2, A–C, and Supplemental Figure 3D).

### Neuronal envelopment is a common phenomenon observed across prion strains.

**Figure 3:**
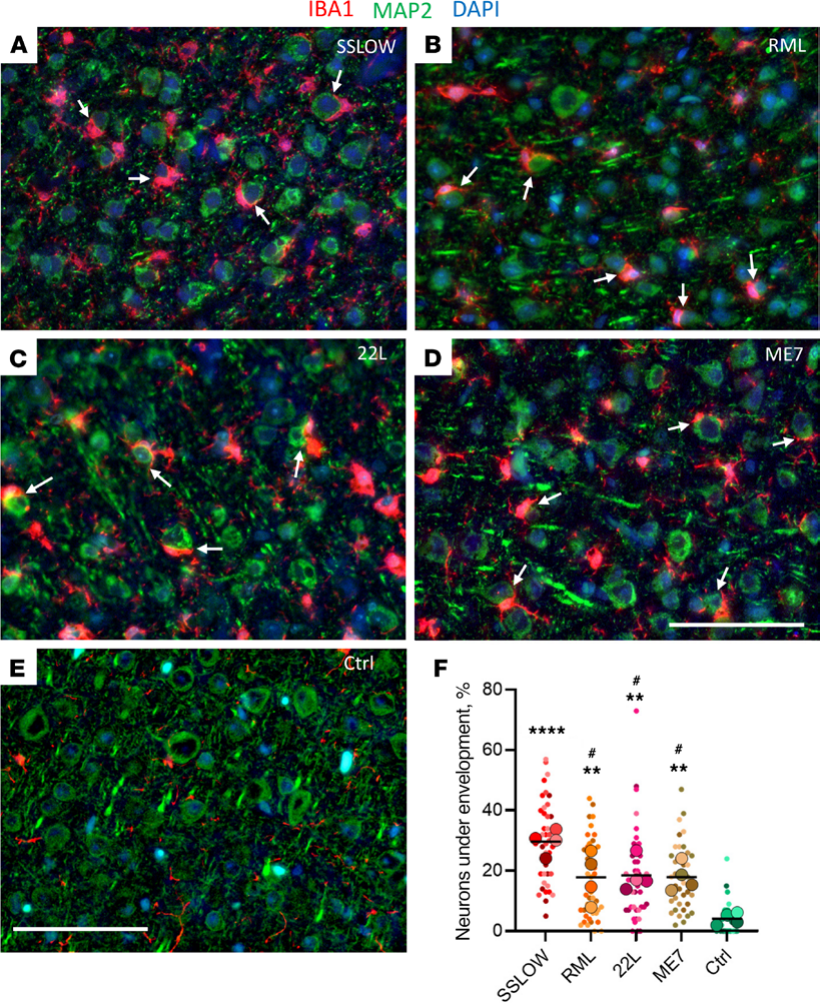
Envelopment of neurons is a common property among prion strains. (**A**–**D**) Representative images of neuronal envelopment (arrows) by reactive microglia in cortex of C57BL/6J mice infected with SSLOW (**A**), RML (**B**), 22L (**C**), and ME7 (**D**) via i.p. and mock-inoculated age-matched mice (**E**) stained using anti-IBA1 (red) and anti-MAP2 (green) antibodies. The dataset from the 22L-infected and mock-inoculated control groups were also used to report microglia-neuronal colocalization in the Figure 5 of the manuscript by Sinha et al. (49). (**F**) Quantification of the percentage of neurons that are undergoing envelopment in cortexes of mice infected with SSLOW, RML, 22L or ME7, and mock-infected control mice (Ctrl). Colors represent different brains. Dots represent individual fields of view. Average values for each brain are shown as circles. Black lines mark strain means. *n* = 4 animals per group. **** *P* < 0.0001; **P < 0.01, statistical significance versus control; ^#^*P* < 0.05, statistical significance versus SSLOW by ordinary 1-way ANOVA followed by Dunnett’s multiple-comparison tests. Scale bars: 50 μm.

C57BL/6J mice, infected i.p. with 4 prion strains (SSLOW 159 ± 13 dpi, RML 209 ± 10 dpi, 22L 216 ± 24 dpi, and ME7 293 ± 32 dpi to terminal disease), showed partial envelopment of neurons by reactive microglia (Figure 3, A–D and F). Notably, by the terminal stage of the disease, SSLOW mice showed the highest percentage of partially encircled neurons. Among the 4 strains, SSLOW was characterized by the shortest incubation time and displayed the most pronounced contacts between microglial soma and neurons (Figure 3, A and F). In the mock-inoculated control group, close contacts between microglial soma and neurons were very rare (Figure 3, E and F).

Mouse-adapted prion strains employed here display distinctive strain-specific cell tropism. For instance, 22L PrP^Sc^ was predominantly associated with astrocytes, while ME7 PrP^Sc^ exhibited a prevalent localization with neurons (39). In the case of RML, the preference of PrP^Sc^ for astrocytes versus neurons is brain region dependent (39). Despite these strain-specific cell tropisms, the observation of neuronal envelopment across all strains, coupled with comparable percentages of envelopment among ME7 (mean 17.9%), 22L (mean 18.1%), and RML (mean 17.1%) strains, irrespective of their cell tropism, implies that the envelopment is not solely driven by PrP^Sc^ association with neurons (Figure 3F).

### The majority of neurons exhibit only partial envelopment.

**Figure 4:**
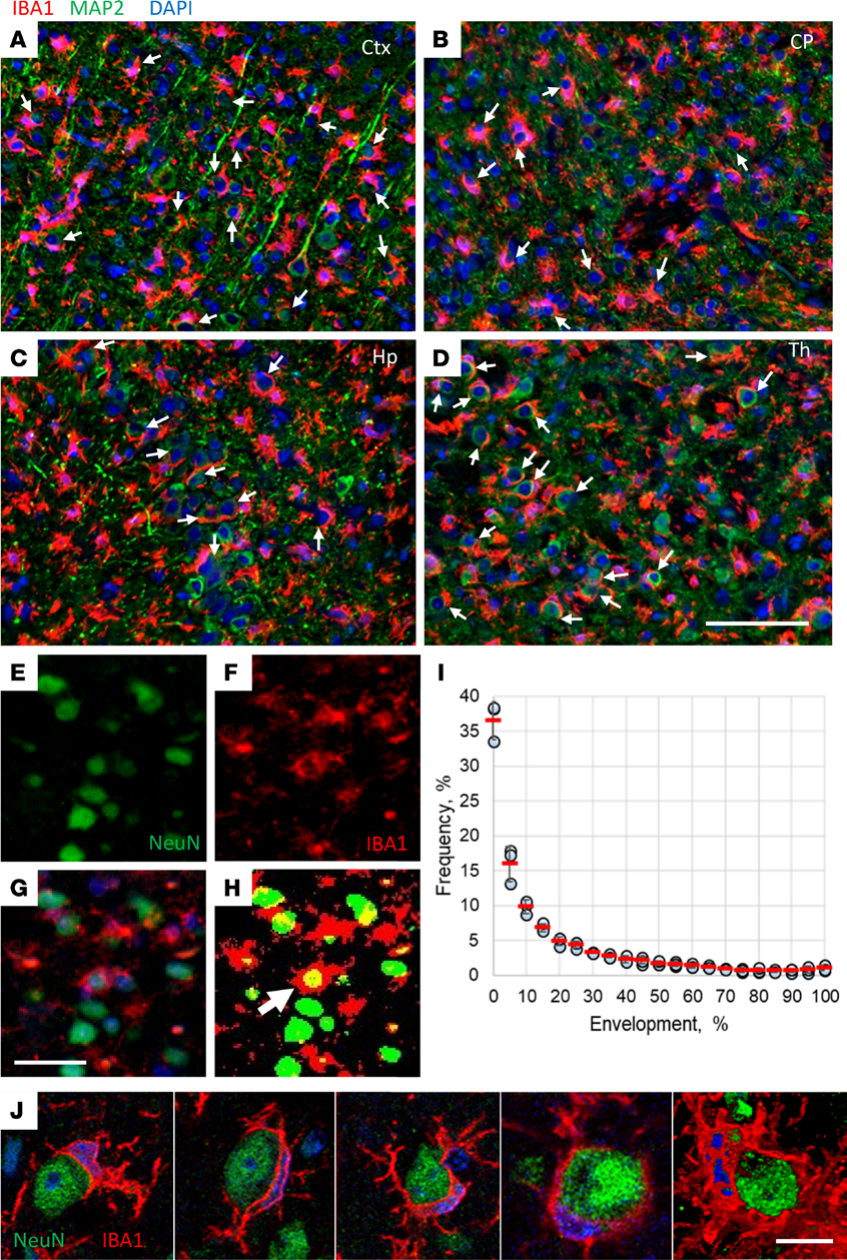
The vast majority of neurons are only partially enveloped. (**A**–**D**) Envelopment of neurons (arrows) in cerebral cortex (Ctx) (**A**), caudate/putamen (CP) (**B**), hippocampus (Hp) (**C**), and thalamus (Th) (**D**) of C57BL/6J mice infected with SSLOW via i.c. route at the terminal stage. Staining was performed using anti-IBA1 (red) and anti-MAP2 (green) antibodies. (**E**–**I**) Quantification of the neuronal area enveloped by microglia. With epifluorescence microscopy, light penetrates the full depth of a cell; thus an overlap of signal from neuronal (NeuN, **E**) and microglial (IBA1, **F**) markers is observed when a neuronal body is undergoing envelopment by a microglial cell (**G**). The percentage of neuronal area enveloped by microglia is estimated for individual neurons as a fraction of NeuN signal overlapped with IBA1 signal. (**H**) Merged thresholds of images from IBA1 and NeuN channels. Arrow points to a rare event of a complete envelopment. (**I**) Frequency distribution of the enveloped areas of the individual neurons quantified for the cortex of SSLOW-infected mice. *n* = 3 animals, *n* = 1,169, 1,422, and 1,551 envelopment events for individual animals. (**J**) A gallery of confocal microscopy images of neurons partially or fully enveloped by microglia. Scale bars: 100 μm (**A**–**D**); 20 μm (**E**–**H**); 5 μm (**J**).

Envelopment of neurons was observed across all brain regions affected by prions, including the cerebral cortex, caudate/putamen (or striatum), hippocampus, and thalamus (Figure 4, A–D). The same brain regions showed spongiform change, PrP^Sc^ accumulation, and reactive gliosis as judged from ionized calcium-binding adaptor molecule 1 (IBA1) and glial fibrillary acidic protein (GFAP) staining (Supplemental Figures 1 and 2), suggesting that neuronal envelopment is linked to prion pathology. Interestingly, fully enveloped neurons were rare, whereas most of the enveloping events were partial.

To estimate the degree of envelopment for individual neuronal cells, we calculated the area of NeuN signal covered by IBA1 signal in the cortex (Figure 4, E–I). Rigorous examination of confocal microscopy *Z*-stack images confirmed that we indeed observed genuine encircling events, rather than mere vertical adjacency of NeuN^+^ and IBA1^+^ cells (Figure 4J and Supplemental Figure 5, A and B, Supplemental Video 3, and Supplemental Video 4). The lack of envelopment of astrocytes argues that envelopment is specific to neurons and is not simply due to microglia proliferation and overcrowding (Supplemental Figure 2B). In the cerebral cortex of SSLOW-infected mice analyzed at the terminal stage, approximately 36% of neurons showed no contact with microglia, while the remaining neurons exhibited varying degrees of encircling by the IBA1^+^ cells (Figure 4I). Intriguingly, the complete encircling of the entire neuronal signal with the IBA1 signal was observed in only a small percentage of neurons (less than 1%) (Figure 4I). Consequently, the majority of neurons were found to be only partially encircled.

### The envelopment of neurons by microglia in a cortex does not lead to a reduction in the total number of neurons.

Previous studies have shown that the phagocytic clearance of a cell by microglia in a mouse brain takes 25 minutes to 2 hours (15, 40). In the terminal stage of prion disease, as few as 15.1% of cortical neurons (for RML) were observed to undergo envelopment (Figure 3F). If 15% or more of the neuronal population are phagocytosed every 2 hours, the entire population would be cleared in less than 12 hours. Consequently, we aimed to establish a timeline for neuronal loss in prion-infected cortex.

**Figure 5:**
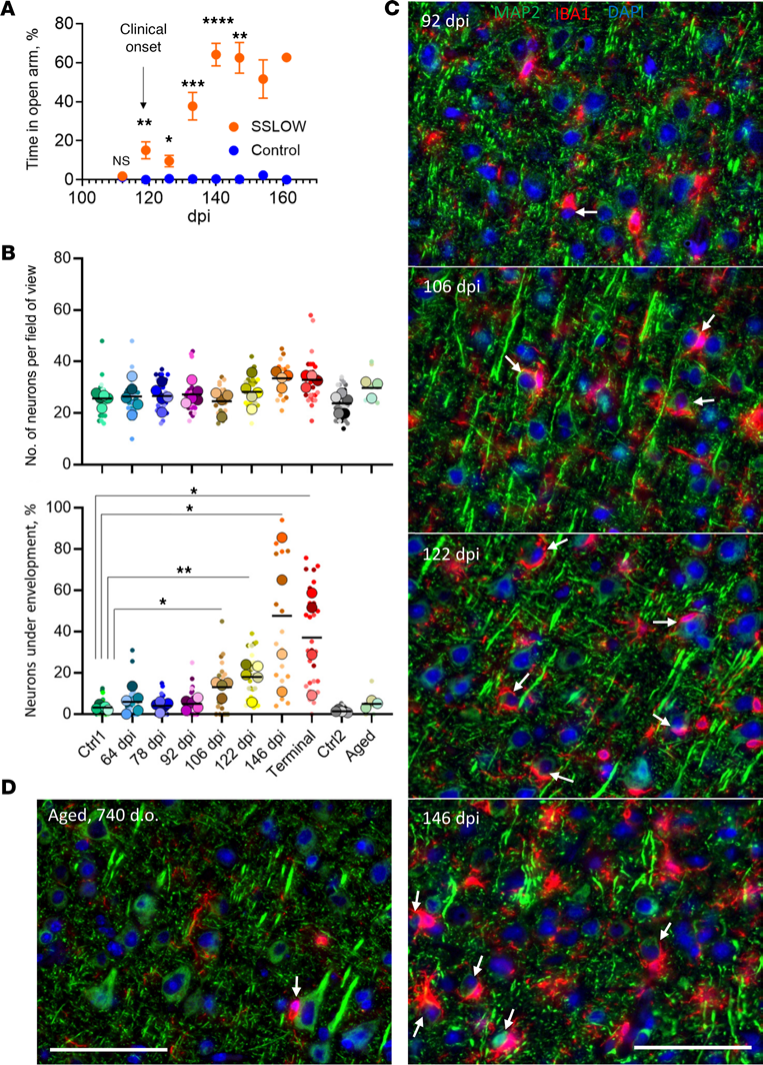
Time course of the envelopment. (**A**) The clinical onset of the disease in C57BL/6J mice infected with SSLOW via i.p. established using the EPM test. Mice were subjected to EPM sessions once per week starting from the preclinical stage. After the first training sessions (not shown), mice naturally acquired a strong preference for the closed arms. The clinical onset was defined as a time point, when the time on open arms consistently increased in comparison with the noninfected age-matched control group (shown by arrow). *n* = 5 for control; *n* = 14 for SSLOW until 119 dpi; *P < 0.05; **P < 0.01; ***P < 0.001; ****P < 0.0001, by Tukey’s multiple-comparison test. (**B**) Change in the total number of MAP2^+^ neurons (upper plot) and the percentage of MAP2^+^ neurons undergoing envelopment (lower plot) during disease progression. Ctrl1 and Ctrl2 are age-matched controls for 64 dpi and terminal, respectively. Aged 740-day-old mice. Colors represent different brains. Dots represent individual fields of view. Average values for each brain are shown as circles. *n* = 3–5 animals per time point. Means are marked by black lines. Comparison of means with Ctrl1 was performed by nonparametric Mann-Whitney *U* test. *P < 0.05; **P < 0.01. (**C**) Representative images of neuronal envelopment in the cerebral cortex of SSLOW-infected C57BL/6J mice collected at 92 dpi, 106 dpi, 122 dpi (disease onset), and 146 dpi. (**D**) Representative images of aged brains (740 days old). Staining with anti-IBA1 (red) and anti-MAP2 (green) antibodies. Arrows point at neurons undergoing envelopment. Scale bars: 50 μm.

In C57BL/6J mice challenged with SSLOW via i.p. route, clinical onset occurred around 120 dpi (Figure 5A). Mice were euthanized at 157–166 dpi, when they showed 20% weight loss along with severe motor impairment and behavior deficits (Figure 5A). Neuronal quantification and the percentage of neurons under envelopment were assessed in cortices at regular time points, starting at 64 dpi. At 64 dpi, 78 dpi, and 92 dpi, the percentage of neurons with extensive body-to-body neuron-microglia cell contacts was comparable to the control group, matched in age to the 64 dpi group (Figure 5, B–D). However, a statistically significant increase in the percentage of neurons undergoing envelopment was noted at 106 dpi (mean 12.4% versus 3.0% in control), 2 weeks prior to clinical onset (Figure 5, B and C). The percentage of neurons undergoing envelopment rose with disease progression, peaking at 146 dpi (mean 44.5%) (Figure 5, B and C). However, despite a substantial number of microtubule associated protein 2–positive (MAP2^+^) neurons being partially enveloped at the clinical stage of the disease, the total number of MAP2^+^ cells in the cortex did not decrease with the disease progression, suggesting that envelopment does not result in neuronal death (Figure 5B). Since mice must be euthanized at 20% weight loss, defined as the endpoint per requirements of the animal care committee, we do not know whether any neuronal loss would occur in cortices after this point and upon reaching the actual terminal stage of the disease.

In a cohort of aged C57BL/6J mice (604–704 days old), the number of neurons undergoing envelopment was comparable to that in 2 control groups, matched in age either to the 64 dpi or 157–166 dpi groups (Figure 5, B and D). This observation suggests that the phenomenon of envelopment is not prominently associated with normal aging.

The analysis of gene expression in SSLOW-inoculated mice revealed that, despite the absence of a decline in the cortical neuronal population, neuron-specific genes were downregulated. Notably, genes associated with ligand-gated ionic channels related to memory and learning (*Gabrg1*, *Grin1*, *Grin2b*, *Grm2*) and synaptic neurotransmission (*Snap25*, *Syn2*, *Syp*) exhibited reduced expression levels (Supplemental Figure 6).

In summary, the above findings establish that neuronal envelopment events were on the rise prior to the clinical onset of the disease. Neuronal envelopment was not accompanied by neuronal loss. These findings are consistent with the fact that, in the majority of cases, the encircling of neuronal soma was only partial.

### The envelopment is not selective toward the subpopulation of the most vulnerable PV^+^ neurons.

In previous studies, GABAergic parvalbumin-positive (PV^+^) neurons were identified as the most vulnerable in prion diseases (41–44). In both humans affected by CJDs and mice infected with mouse-adapted strains, a decline in PV^+^ neurons was observed at the subclinical stage of the disease (41–44). If microglia are responsible for the loss by selectively targeting PV^+^ neurons, we would expect PV^+^ neurons to be enveloped early, i.e., prior to clinical onset. Contrary to this hypothesis, statistically significant envelopment of PV^+^ neurons was observed only at terminal time points (Supplemental Figure 7, A and B). Moreover, both PV^+^ and PV^–^ neurons were subject to envelopment, arguing that this process is not selective toward PV^+^ neurons (Supplemental Figure 7A).

### Neurons under envelopment lack apoptotic markers.

**Figure 6:**
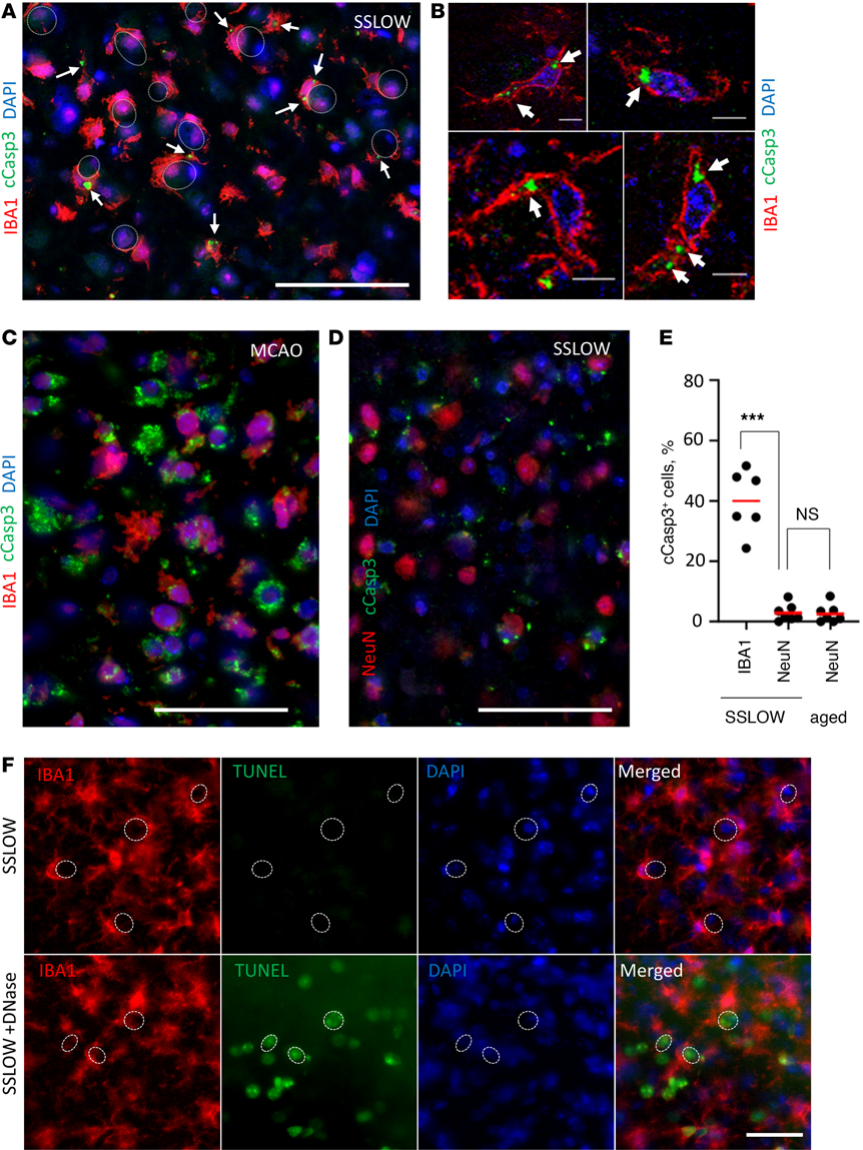
Neurons undergoing envelopment lack apoptotic markers. (**A**–**C**) Coimmunostaining of SSLOW-infected C57BL/6J mice at the terminal stage (**A** and **B**) or C57BL/6J mice subjected to MCAO and analyzed 5 days after insult (**C**) using antibody to activated cCasp3 and anti-IBA1 antibody. (**B**) Confocal microscopy imaging illustrates the intracellular localization of cCasp3 (pointed by arrows) in microglia of SSLOW-infected mice. (**D**) Coimmunostaining of SSLOW-infected C57BL/6J mice using anti-cCasp3 and anti-NeuN antibodies. (**E**) Percentage of cCasp3^+^ microglia (IBA1) and neurons (NeuN) in cortexes of SSLOW-infected mice and in neurons of aged 607- to 740-day-old C57BL/6J mice. ***P < 0.001, Brown-Forsythe and Welch’s ANOVA followed by Dunnett’s T3 multiple-comparison tests. *n* = 6–7 fields of view. (**F**) TUNEL staining of SSLOW-infected C57BL/6J mouse at the terminal stage, and sections from the same mouse pretreated with DNase and used as positive controls. Dashed circular lines represent examples of neuronal envelopment. Scale bars: 50 μm (**A**, **C**, and **D**); 5 μm (**B**); 100 μm (**F**).

To investigate whether neurons that undergo envelopment remain viable, we assessed the presence of apoptotic markers. In previous studies on adult neurogenesis through apoptosis-coupled phagocytosis, the neuronal cells at the early stages of phagocytosis by microglia, i.e., the stage of partial engulfment, displayed the strongest activated (cleaved) caspase-3 signal (cCasp3) (15). Staining terminal SSLOW-infected C57BL/6J mice with an antibody to cCasp3, an early apoptosis marker, revealed that the majority of neurons under envelopment were cCasp3 negative (Figure 6, A, D, and E). In fact, the number of cCasp3^+^ neurons was low and comparable to that of normally aged mice (Figure 6E). On the contrary, in positive controls, C57BL/6J mice subjected to ischemia showed extensive neuronal cCasp3 staining (Figure 6C).

Interestingly, in prion-affected brains, reactive microglia exhibited cCasp3-positive puncta (Figure 6, A, B, and E). Confocal microscopy imaging confirmed that cCasp3 immunoreactivity was associated with microglia and displayed intracellular localization (Figure 6B). Caspase-3 activation in microglia has been recognized as a switch between proinflammatory activation and cell death, as observed in neurodegenerative diseases including Alzheimer’s and Parkinson’s diseases (45, 46).

As an alternative approach for identifying apoptotic cells, we employed the TUNEL assay. TUNEL stains apoptotic cells by detecting DNA fragmentation. In the cortices of terminal SSLOW-infected mice, neurons undergoing engulfment were negative for TUNEL staining (Figure 6F). However, pretreatment of brain slices from the same mice with DNase revealed DNA fragmentation detected by the TUNEL staining (Figure 6F). In summary, these results indicate that neurons undergoing envelopment by microglia lack apoptotic markers.

### Changes in neuronal functional state precede their envelopment.

**Figure 7:**
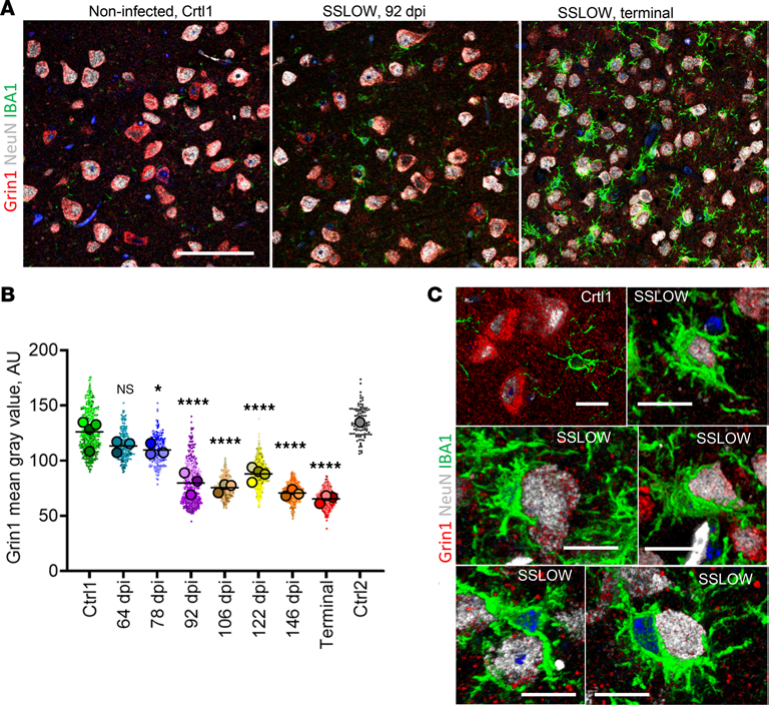
A decline in neuronal levels of Grin1 with disease progression. (**A**) Confocal microscopy images of C57BL/6J mice infected with SSLOW via i.p. examined at 92 dpi and terminal stage (157–166 dpi) using anti-Grin1 (red), anti-NeuN (gray), and anti-IBA1(red) antibodies. (**B**) Quantification of Grin1 levels during disease progression. Colors represent different brains. Grin1 mean intensity values in individual neurons are shown as dots. Average values for each brain are shown as circles. Black lines mark time-point means. The time-point data were compared with Ctrl1 (age-matched controls for 64 dpi mice) by ordinary 1-way ANOVA followed by Dunnett’s multiple-comparison tests. *P < 0.05; ****P < 0.0001. *n* = 3–4 brains per time point. Age-matched control for terminal mice (Ctrl2) is provided as a reference. (**C**) Confocal microscopy 3D reconstruction images of individual neurons in age-matched control and SSLOW-infected C57BL/6J mice illustrating low Grin1 signal in neurons enveloped by microglia. Scale bars: 50 μm (**A**); 10 μm (**C**).

Transcriptome analysis identified several neuron-specific genes that were markedly downregulated at the terminal stage. Among these was *Grin1*, which encodes a critical subunit of the *N*-methyl-d-aspartate (NMDA) receptor, essential for synaptic plasticity, memory, and learning. To test whether neuronal changes precede their envelopment by microglia, Grin1 levels were quantified in individual cortical neurons at regular time points using confocal microscopy (Figure 7A). A modest yet statistically significant drop in Grin1 signal was observed at 78 dpi, followed by a steeper decline at 92 dpi (Figure 7B). This decline suggests alterations in the neuronal functional state, indicative of neuronal stress. Notably, these changes occurred before the onset of neuronal envelopment by microglia, which begins at 106 dpi (Figure 5B). Confocal microscopy confirmed that microglia envelop neurons with reduced Grin1 signal (Figure 7C).

### Neurons are enveloped by PrP^Sc^-positive microglia.

**Figure 8:**
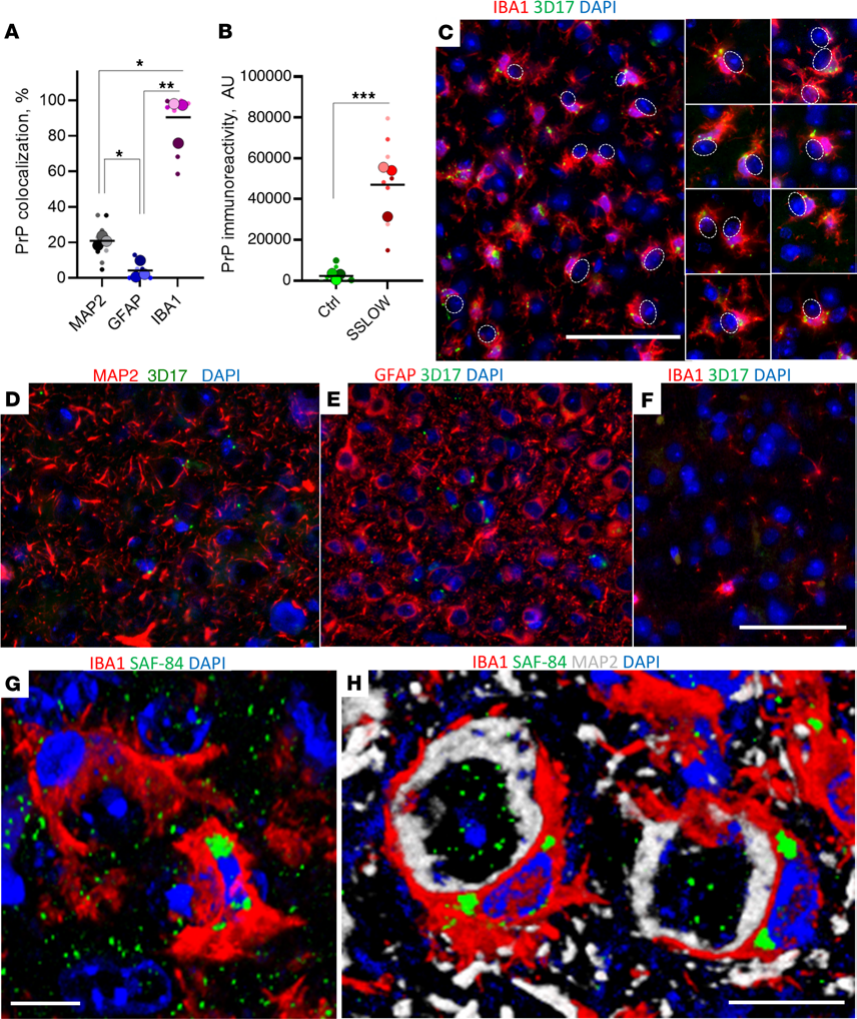
PrP^Sc^ colocalizes with reactive microglia. (**A**) Quantification of PrP^Sc^ colocalization with MAP2^+^, GFAP^+^, and IBA1^+^ cells in SSLOW-infected C57BL/6J mice analyzed at the terminal stage. *P < 0.05; **P < 0.01 by Brown-Forsythe and Welch’s ANOVA followed by Dunnett’s T3 multiple-comparison tests.(**B**) Quantification of PrP immunoreactivity in SSLOW-infected and age-matched control C57BL/6J mice. Colors represent different brains. Dots represent individual values for field of view. Average values for each brain are shown as circles. Black lines mark group means. *n* = 3 animals per group. ***P < 0.001, unpaired *t* test with Welch’s correction. (**C**–**F**) Representative images of SSLOW-infected C57BL/6J at the terminal stage (**C**–**E**) and age-matched control mice (**F**) coimmunostained using anti-PrP (3D17) and anti-IBA1 (**C** and **F**), anti-MAP2 (**D**), or anti-GFAP antibodies (**E**). In **C**, dashed circles show neuronal envelopment; a gallery of images on the right shows PrP^Sc+^ microglia that envelop neurons. (**G** and **H**) 3D reconstruction of confocal microscopy images of PrP^Sc+^ microglia enveloping neurons in SSLOW-infected C57BL/6J mice at terminal stage. Staining using anti-PrP (SAF-84) (**G** and **H**), anti-IBA1 (**G** and **H**), and anti-MAP2 antibodies (**H**). Scale bars: 50 μm (**C**–**F**); 5 μm (**G** and **H**).

Reactive microglia may target viable neurons because they sense the accumulation of prion aggregates on neuronal surfaces. To test this, brains of SSLOW-infected C57BL/6J mice were coimmunostained using anti-PrP antibody 3D17 in combination with anti-MAP2, anti-IBA1, or anti-GFAP antibodies. To avoid confusion between diffuse PrP^Sc^ and PrP^C^, fluorescence microscopy imaging was optimized to preferentially detect granular PrP^Sc^ deposits, characterized by a bright emission. In SSLOW-infected animals, the majority of granular PrP immunoreactivity was associated with reactive microglia, including the microglia that enveloped neurons (Figure 8, A and C). Noninfected, age-matched controls did not display any granular PrP immunoreactivity, arguing that in prion-infected animals, the granular PrP signal is attributed to PrP^Sc^ (Figure 8, B and F). In SSLOW-infected mice, only a subtle granular PrP signal was detected in neurons (Figure 8, A and D). Very minimal, if any, PrP immunoreactivity was detected in astrocytes (Figure 8, A and E). A similar pattern of granular PrP immunoreactivity, the majority of which colocalized with microglia, was observed in a parallel staining using different anti-PrP antibody SAF-84 (Supplemental Figure 8). Unlike neurons or astrocytes, microglia do not replicate PrP^Sc^ but can acquire PrP^Sc^ positivity via phagocytic uptake (26, 27).

Three-dimensional reconstruction of confocal microscopy imaging confirmed the intracellular localization of SSLOW PrP^Sc^ aggregates in reactive microglia (Figure 8G). Furthermore, triple coimmunostaining using anti-MAP2 and anti-IBA1 antibodies revealed that PrP^Sc^ aggregates in microglia enveloping neurons localized to perinuclear sites (Figure 8H, Supplemental Figure 9, and Supplemental Video 5). Confocal microscopy *Z*-stack video demonstrated that PrP^Sc^ deposits occupy substantial cell volume in microglia enveloping neurons (Supplemental Figure 9 and Supplemental Video 5).

In mice infected with 22L, granular PrP^Sc^ was also found in association with microglia (Supplemental Figure 10A), though it appeared to be less abundant. In microglial cells enveloping neurons, 22L and SSLOW PrP^Sc^ showed similar patterns of intracellular localization, as observed through 3D reconstruction of confocal microscopy images (Supplemental Figure 10B). The lower abundance and smaller size of granular 22L PrP^Sc^ compared with SSLOW PrP^Sc^ could be attributed to reduced proteolytic stability and a higher rate of clearance of 22L in lysosomes.

### Phagocytic uptake of PrP^Sc^ by microglia precedes neuronal envelopment.

**Figure 9:**
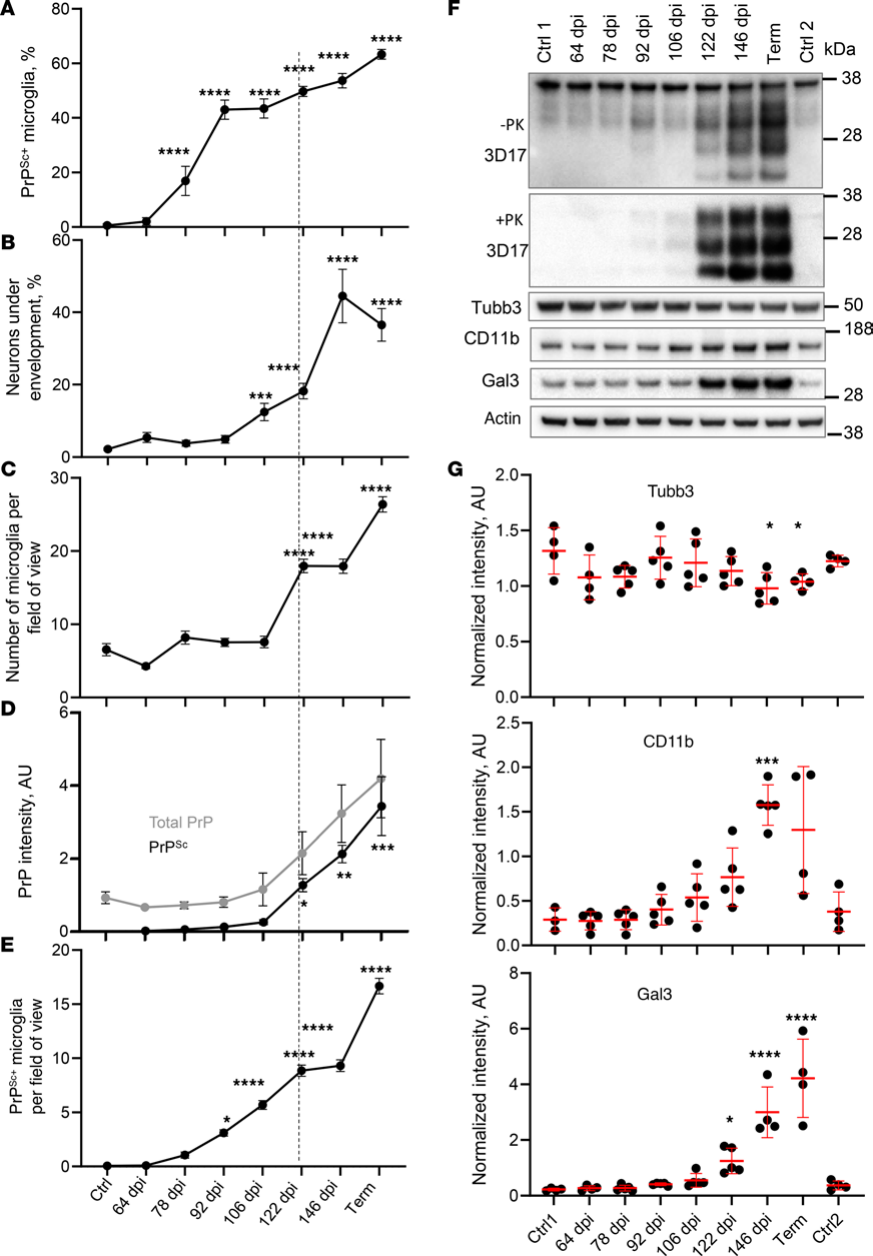
Time course of PrP^Sc^ uptake, neuronal envelopment, and microglia proliferation. Changes in the percentage of PrP^Sc+^ microglia (**A**), the percentage of MAP2^+^ neurons under envelopment (**B**), the total number of IBA1^+^ microglial cells per field of view (**C**), the amounts of total PrP (no PK treatment) and PrP^Sc^ (after PK treatment) as estimated by Western blot (**D**), and the total number of PrP^Sc+^ microglia per field of view (**E**) in C57BL/6J mice infected with SSLOW via i.p. route with disease progression. Data are represented as mean ± SEM. Comparisons with Ctrl (combined age-matched controls for 64 dpi and terminal points) were done using Kruskal-Wallis test followed by Dunn’s multiple-comparisons test. *P < 0.05; **P < 0.01; ***P < 0.001; ****P < 0.0001. *n* = 18–63 fields of view (3–5 brains) per time point for **B**, and *n* = 17–60 (4–5 brains) for **A**, **C**, and **E**. Term, terminal animals collected at 157–166 dpi. Dashed line shows clinical onset. (**F**) Representative Western blots of total PrP (no PK treatment, -PK), PrP^Sc^ (after PK treatment, +PK), Tubb3, CD11b, and Gal3 in brains of SSLOW-infected C57BL/6J mice. PrP is detected by 3D17 antibody. (**G**) Quantification of Western blots of Tubb3, CD11b, and Gal3; signal intensities were normalized per intensities of actin for each individual Western blot. In **F** and **G**, Ctrl1 and Ctrl2 are age-matched controls for 64 dpi and terminal time points, respectively. Data are represented as means ± SD. *n* = 3–5 animals per group, *P < 0.05; ***P < 0.001; **** *P* < 0.0001; each time point was compared with the combined control group (Ctrl1 + Ctrl2) by Brown-Forsythe and Welch’s ANOVA followed by Dunnett’s T3 multiple-comparison tests.

The observation of PrP^Sc^ deposits in reactive microglia raises the possibility that microglia phagocytically uptake PrP^Sc^ prior to engaging in neuronal envelopment. To test this hypothesis, we quantified the percentage of PrP^Sc+^ microglial cells and the percentage of neurons undergoing envelopment in the cortices of C57BL/6J mice at regular time points starting at 64 dpi. A significant increase in the percentage of PrP^Sc+^ microglia was observed at 78 dpi (Figure 9A and Supplemental Figure 11), whereas envelopment became noticeable only from 106 dpi onward (Figure 9B). At 106 dpi, nearly 50% of microglial cells showed well-detectable PrP^Sc^ deposits (Figure 9A). Remarkably, despite a substantial increase in the percentage of PrP^Sc+^ microglia, the total amount of PrP^Sc^ in a crude brain, as quantified by Western blot, remained at very low levels until 106 dpi (Figure 9D). These findings suggest that microglia manage to control prion replication through the removal of PrP^Sc^ via phagocytic uptake. Notably, a substantial increase in the number of IBA1^+^ cells was observed after 106 dpi (Figure 9C), tripling their phagocytic capacity. While infiltration of myeloid cells from the periphery cannot be entirely excluded, previous studies have established that the expansion of microglia in prion diseases is primarily due to the proliferation of resident myeloid cells, with minimal recruitment of peripheral myeloid cells (34, 47). Consistent with a substantial boost in phagocytic capacity of IBA1^+^ cells was an increase in CD11b and Gal3 levels observed during the clinical stage (Figure 9, F and G). CD11b and Gal3 are involved in 2 different phagocytic pathways. Notably, the percentage of PrP^Sc+^ microglia continued to rise even after 106 dpi (Figure 9A) alongside their substantial proliferation (Figure 9C), indicating that proliferated cells were actively engaged in the phagocytic uptake of PrP^Sc^ (Figure 9D). However, despite the profound boost in phagocytic capacity attributed to proliferation, prion replication spun out of control after 106 dpi (Figure 9, D and F). This time point coincides with the rise of microglial envelopment of neurons, which only intensifies with disease progression (Figure 9B). To summarize, phagocytic uptake of PrP^Sc^ by microglia preceded neuronal envelopment by 4 weeks, whereas clinical onset followed neuronal envelopment.

### Microglia engaged in envelopment are characterized by activated hypertrophic lysosomes.

**Figure 10:**
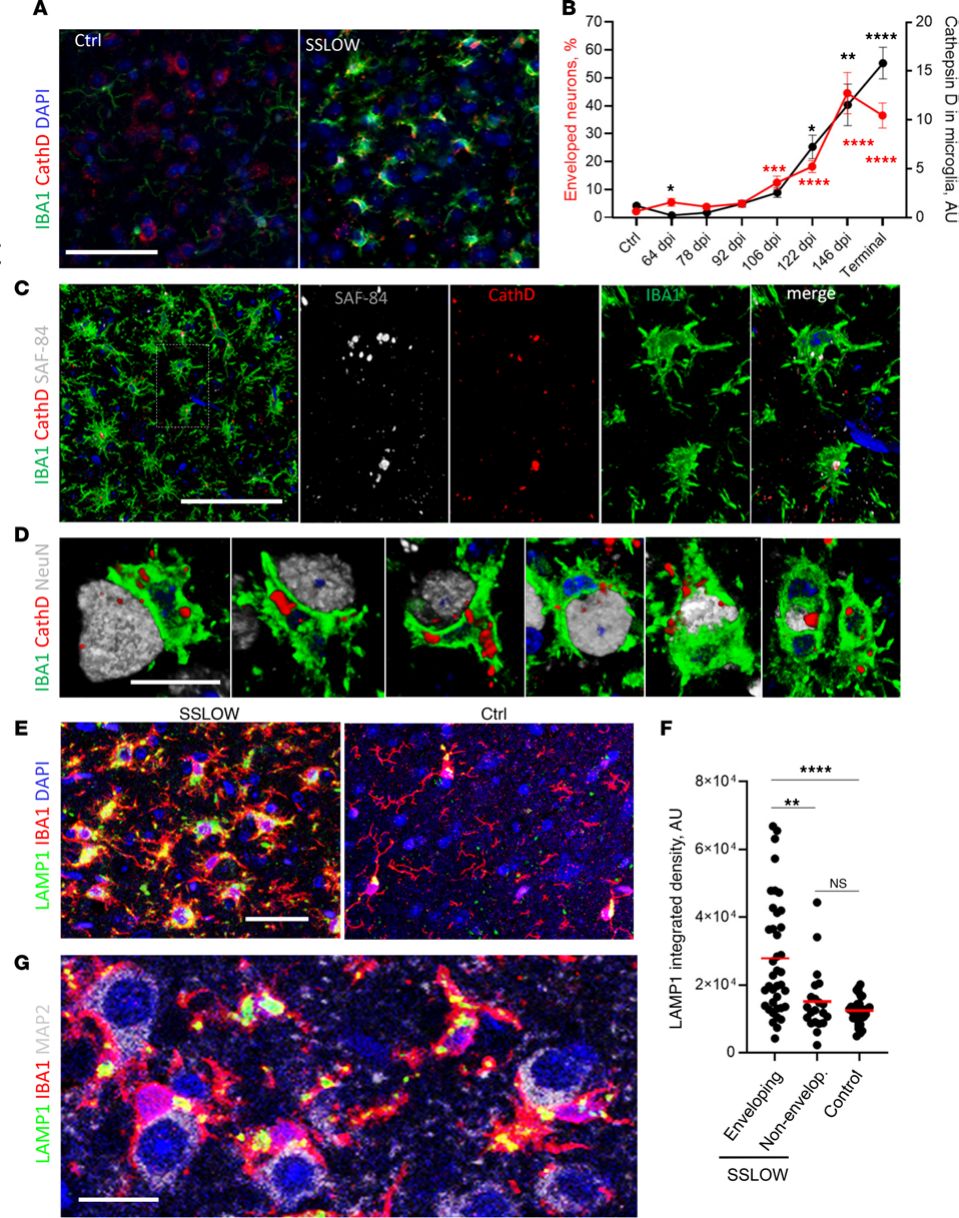
Microglia engaged in envelopment have activated hypertrophic lysosomes. (**A**) Epifluorescence microscopy images of cortices of noninfected age-matched control and C57BL/6J mice infected with SSLOW via i.p route and examined at the terminal stage using anti–cathepsin D (red) and anti-IBA1 (green) antibodies. (**B**) Changes in the integrated density of cathepsin D associated with microglia (black) and the percentage of MAP2^+^ neurons undergoing envelopment (red) with disease progression. Data are represented as mean ± SEM. *n* = 11–63 fields of view (3–6 brains) per time point. Comparisons to Ctrl (age-matched control for 64 dpi and terminal points) were done using Kruskal-Wallis test followed by Dunn’s multiple-comparisons test. *P < 0.05; **P < 0.01; ***P < 0.001; ****P < 0.0001. Terminal animals collected at 157–166 dpi. (**C** and **D**) 3D reconstruction of confocal microscopy imaging of SSLOW-infected C57BL/6J mice illustrating colocalization of PrP^Sc^ (SAF-84, gray) with cathepsin D (red) in microglia (IBA1, green) (**C**) and envelopment of neurons (NeuN, gray) by cathepsin D–positive (red) microglia (IBA1, green) (**D**). (**E**) Maximum intensity projection confocal images of LAMP1^+^ compartments (green) in microglia (IBA1) in cortices of SSLOW-infected C57BL/6J mice analyzed at the terminal stage along with age-matched control mice. (**F**) Quantification of LAMP1 integrated density in individual microglial cells engaged or not engaged in neuronal envelopment in cortices of SSLOW-infected mice, and age-matched controls. *n* = 21–39 individual cells. **P < 0.01; ****P < 0.001, by Kruskal-Wallis test followed by Dunn’s multiple-comparisons test. (**G**) Confocal microscopy image of LAMP1^+^ compartments in cortices of SSLOW-infected C57BL/6J mice. Scale bars: 50 μm (**A** and **C**); 20 μm (**G**); 10 μm (**D** and **G**).

The observation of PrP^Sc^-positive microglia aligns well with previous studies that document profound upregulation of phagocytic activity in microglia during prion diseases (48–50). Given that sustained phagocytic activity necessitates the upregulation of lysosomal degradation, we quantified cathepsin D, a proteolytic enzyme used to assess lysosomal activity, during disease progression (36). In noninfected mice, the majority of cathepsin D immunoreactivity was not associated with IBA1^+^ cells (Figure 10A). However, in SSLOW-infected mice, granular cathepsin D deposits were observed in microglia (Figure 10A). The upregulation of microglia-associated cathepsin D coincided with a rise in neuronal envelopment (Figure 10B), with both processes progressing in parallel with the disease advancement. Confocal microscopy revealed colocalization of cathepsin D with PrP^Sc^ deposit in reactive microglia (Figure 10C). Careful examination of individual envelopment events revealed cathepsin D–positive microglia engaged in encircling neurons (Figure 10D). Moreover, cathepsin D–positive vacuoles were observed on the periphery of neuronal material that was fully engulfed by microglia (Figure 10D).

Next, we quantified lysosomal compartments in individual microglial cells by confocal microscopy imaging of lysosome associated membrane protein 1 (LAMP1), which is highly expressed in lysosomal membranes. In contrast to age-matched controls, in SSLOW-infected animals, we observed substantial LAMP1 immunoreactivity associated with microglia (Figure 10E). Remarkably, within the SSLOW-infected brains, LAMP1 immunoreactivity was significantly higher in microglial cells engaged in neuronal envelopment compared with microglia not involved in envelopment (Figure 10, F and G). Collectively, these results support the idea that microglial cells engaged in neuronal envelopment have activated, hypertrophic lysosomes.

### Neuronal envelopment is independent of the CD11b pathway.

**Figure 11:**
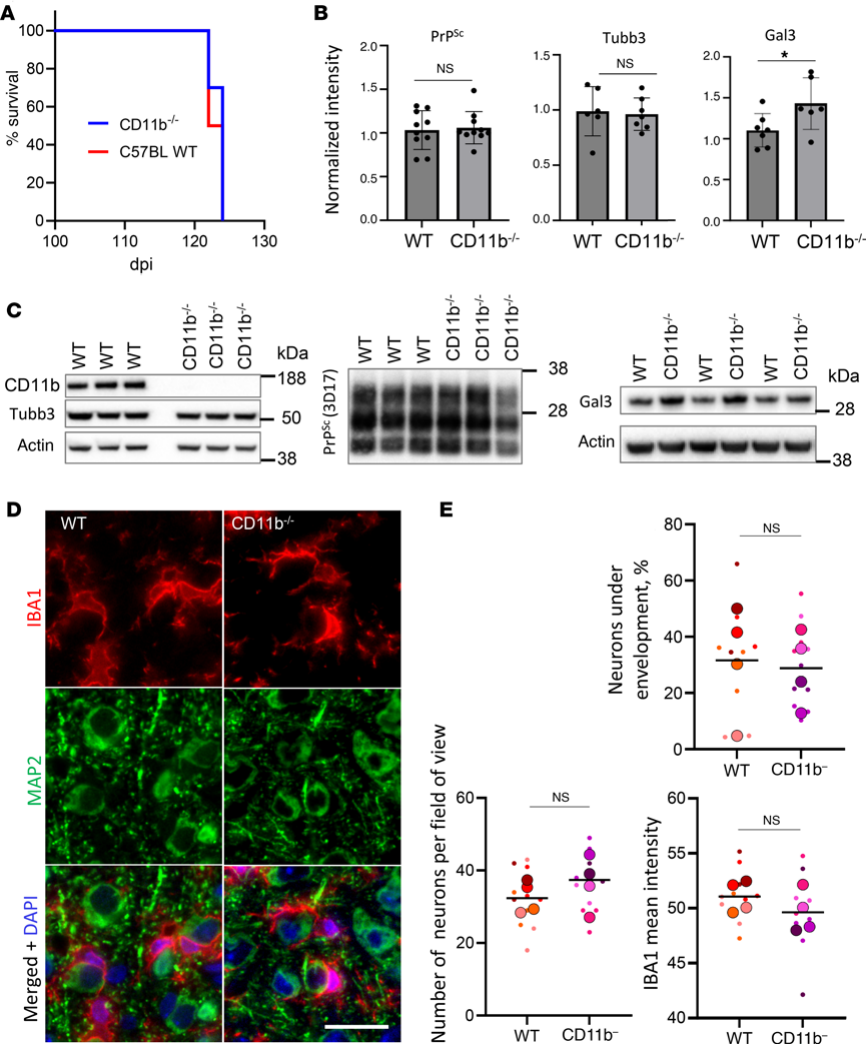
Analysis of neuronal envelopment in CD11b^–/–^ mice. (**A**) Incubation time to terminal disease in CD11b^–/–^ and C57BL/6J control mice (WT) inoculated with SSLOW via i.c. route. *n* = 10 animals per group. Mantel-Cox test of survival curves indicated no significant difference between the groups. (**B**) Densitometric quantification of Western blots for PrP^Sc^, Tubb3, and Gal3 in SSLOW-infected CD11b^–/–^ and WT mice. Data are represented as means ± SD. *n* = 6–10 per group. ^*^P < 0.05, by 2-tailed, unpaired Student’s *t* test. (**C**) Representative Western blots of selected markers in CD11b^–/–^ and WT mice at the terminal stage. For analysis of PrP^Sc^, BHs were digested with PK and stained with 3D17 antibody. (**D**) Envelopment of neurons by microglia in the cortex of SSLOW-infected CD11b^–/–^ and WT mice at the terminal stage stained using anti-IBA1 (red) and anti-MAP2 (green) antibodies. (**E**) Percentage of MAP2^+^ neurons undergoing envelopment, the total number of MAP2^+^ neurons, and IBA1 immunoreactivity in SSLOW-infected CD11b^–/–^ and WT mice at terminal stages. *n* = 4 animals per group. Colors represent different brains. Dots represent individual values. Average values for each brain are shown as circles. Means are marked by black lines. Scale bar: 20 μm.

Among several microglial phagocytic pathways, the CD11b-dependent pathway was previously found to be responsible for phagocytosis of newborn cells, including neurons during development (37, 51). CD11b was also shown to be involved in the phagocytosis of neurons in glia-neuronal cocultures (52). To test whether the same pathway is responsible for envelopment in prion-infected mice, we analyzed the time course of the disease and neuronal envelopment in CD11b-knockout mice infected with the SSLOW strain (Figure 11). There were no differences with respect to incubation time to the terminal disease or the amount of PrP^Sc^ between CD11b^–/–^ and control C57BL/6J (WT) groups (Figure 11, A–C). Neurons were undergoing envelopment in both CD11b^–/–^ and control groups (Figure 11D), while the percentage of enveloped neurons was the same in the 2 groups (Figure 11E). Moreover, no differences between CD11b^–/–^ and the control WT group were found with respect to the density of MAP2^+^ neurons (Figure 11E), the level of expression of neuron-specific protein Tubb3 (Figure 11, B and C), or microglia activation, as judged from IBA1 immunoreactivity (Figure 11E). Modest upregulation of galectin 3 (Gal3) was seen in the CD11b^–/–^ versus the control group (Figure 11, B and C). Gal3, which is involved in alternative phagocytic pathways, is released by activated myeloid cells and acts as an opsonin by binding galactose residues on the cell surface.

### Neuronal envelopment in sCJD individuals.

**Figure 12:**
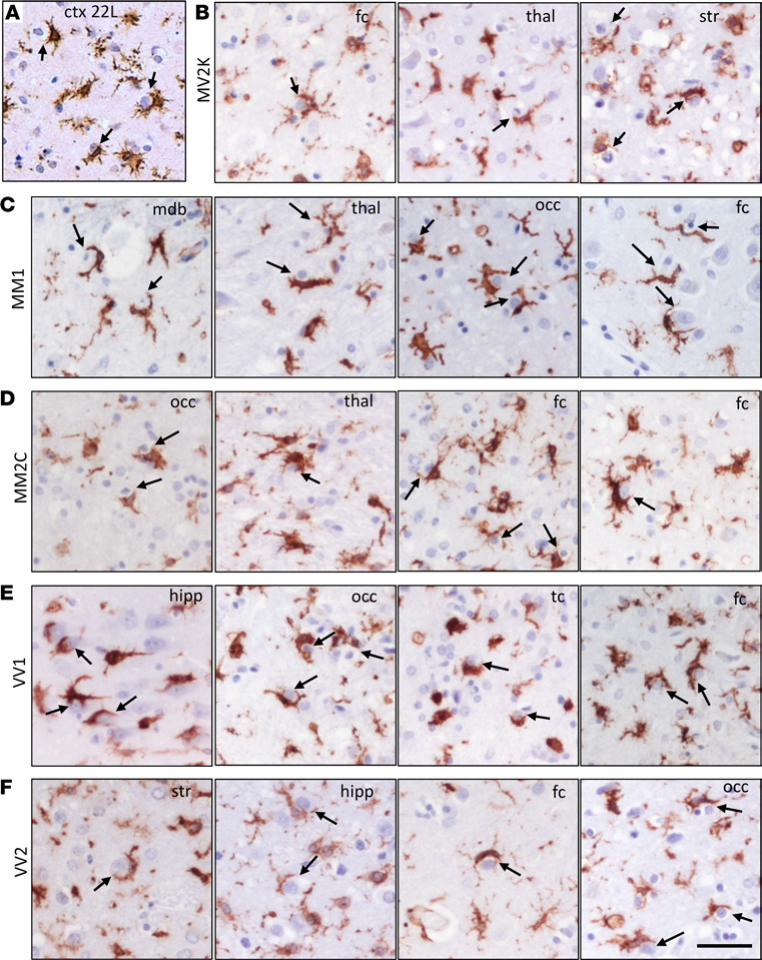
Partial envelopment by reactive microglia in sCJD. (**A**) Representative image of reactive microglia stained with anti-IBA1 antibody in the cortex (ctx) of 22L-infected C57BL/6J mice provided as a reference. (**B**–**F**) Representative images of reactive microglia strained with anti-HLA-DR+DP+DQ (CR3/43) antibody in the following subtypes of sCJD: MV2K (**B**), MM1 (**C**), MM2C (**D**), VV1 (**E**), and VV2 (**F**). Fc, frontal cortex; thal, thalamus; str, striatum; mdb, midbrain; occ, occipital cortex; hipp, hippocampus; tc, temporal cortex. Arrows point at microglia engaged in envelopment. Scale bar: 20 μm.

To check whether the partial envelopment phenomenon occurs in human prion diseases, we examined sCJD brains using staining for the β-chain of human HLA-DR, a marker of reactive microglia. In all examined sCJD subtypes, including MM1, MM2C, VV1, VV2, and MV2K (53), we found reactive microglia engaged in partial envelopment (Figure 12). Akin to prion-infected mouse brains (Figure 12A), such partial envelopment was detected across different human brain regions affected by prions, including frontal and temporal cortex, thalamus, striatum, midbrain, and hippocampus (Figure 12, B–F).

## Discussion

The present study shows that in prion disease, reactive microglia and neurons establish extensive body-to-body contacts resembling cell engulfment, which we refer to as neuronal encircling or enveloping. The phenomenon of envelopment manifested in all brain regions affected by prions. It was consistently observed across mouse-adapted strains in mice and in different subtypes of sCJD in humans. In most microglia-neuronal interaction events, only a portion of the neuronal body was encircled by microglia, and only a small percentage of these contacts progressed to a complete engulfment of the neuronal soma.

The present study provides important insight into the timeline of disease pathogenesis. The enhanced stability of SSLOW PrP^Sc^ within the phagocytic compartments of microglia, compared with PrP^Sc^ from other strains, provides unique opportunities for quantitatively assessing PrP^Sc^ phagocytosis in the brain (54). During the preclinical stage, microglia appear to control prion replication via phagocytic uptake of PrP^Sc^. Notably, the percentage of PrP^Sc+^ microglial cells increases rapidly during the preclinical stage (Figure 9A), when the total amount of PrP^Sc^ still remains at low levels (Figure 9, D and F). This dynamic suggests that the potential accumulation of PrP^Sc^ resulting from replication is counteracted by the phagocytosis of PrP^Sc^. The proliferation of microglia, particularly evident after 106 dpi, triples their phagocytic capacity (Figure 9C). Notably, the percentage of PrP^Sc+^ microglia continues to rise even after 106 dpi (Figure 9A) in conjunction with their active proliferation (Figure 9C), suggesting that proliferated cells are competent in the phagocytic uptake of PrP^Sc^. However, despite the considerable increase in the total phagocytic capacity (Figure 9E), the rate of PrP^Sc^ replication surpasses the rate of its clearance by microglia, leading to a steady buildup of PrP^Sc^ after 106 dpi (Figure 9, D and F).

Remarkably, the late preclinical stage, spanning from 92 to 106 dpi, marks a critical time window when the phagocytic uptake of PrP^Sc^ slows down. Instead, microglia shift their focus and engage in establishing extensive body-to-body neuron-microglia cell contacts (Figure 9B). The onset of encircling coincides with the activation of microglial lysosomes, as monitored by cathepsin D (Figure 10B). Observation of cathepsin D signal on the periphery of neuronal material that was fully engulfed suggests that microglia are prepared for digestion (Figure 10D). Notably, microglial cells engaged in neuronal envelopment were PrP^Sc^ positive (Figure 8H). Consistent with the hypothesis that uptake of PrP^Sc^ upregulates microglial phagocytic activity are recent studies demonstrating that microglia are activated directly by PrP^Sc^ (55) and that phagocytic activity is substantially upregulated in microglia isolated from prion-infected animals (49).

The current study indicates that the uptake of PrP^Sc^ by microglia begins prior to the stage of neuronal encircling, whereas clinical onset follows this process. Previous research has documented the accumulation of PrP^Sc^ not only in association with various cell types but also within intercellular spaces (39). Furthermore, the formation of prion plaques or the deposition of PrP^Sc^ around blood vessels, observed in some prion strains, suggests that PrP^Sc^ can indeed detach from the cell surface (56–58). It remains unclear whether, prior to the stage of neuronal encircling, microglia uptake PrP^Sc^ directly from cell surfaces or after PrP^Sc^ detaches from cells.

Do neurons exhibit any cues that trigger extensive body-to-body neuron-microglia interactions? The hypothesis that reactive microglia selectively target GABAergic, PV^+^ neurons — previously identified as the most vulnerable to prion infection (41–44) — was not supported by our experimental results. The subpopulation of PV^+^ neurons was not among the earliest targets by microglia. In the later stages of the disease, they were under envelopment along with PV^–^ neurons. Microglia could target neurons because the phagocytes sense PrP^Sc^ on neuronal surfaces. In mice infected with SSLOW, the majority of granular PrP^Sc^ was detected in microglia, as seen by fluorescence microscopy. 3,3′-Diaminobenzidine (DAB) staining revealed a substantially wider distribution of diffuse PrP^Sc^ (Supplemental Figure 1). Unlike microglia, neurons do express PrP^C^. In fluorescence microscopy, attributing neuronal PrP immunoreactivity in individual cells exclusively to PrP^Sc^ or PrP^C^ is challenging; therefore, it is difficult to prove or dismiss the hypothesis of neuronal PrP^Sc^ as a cue for microglia. Nevertheless, the fact that neuronal envelopment is shared between different mouse-adapted strains (Figure 3), regardless of their preferential cell tropism, suggests that the presence of PrP^Sc^ on the neuronal surface might not be the major factor driving envelopment. In fact, in mice infected with ME7, PrP^Sc^ is reported to be predominantly associated with neurons, whereas in 22L-infected mice, the majority of PrP^Sc^ is associated with astrocytes (39). Yet the percentage of neuronal engulfment was the same in these 2 strains. Notably, in 22L-infected mice, granular PrP^Sc^ was also associated with microglia (Supplemental Figure 10). Regardless of whether neuronal PrP^Sc^ acts as a signal to attract microglia, the latter might sense other cues indicating neuronal stress or a decline in their functions. Consistent with this hypothesis, a decline in neuronal functions, as evidenced by the downregulation of Grin1, preceded neuron-microglia interactions (Figure 7). The third hypothesis proposes that neurons enter apoptotic cell death, and apoptosis-associated cues activate an eat-me signaling in microglia, resulting in envelopment. However, the absence of activated caspase-3, a marker of early apoptosis, and TUNEL staining in neurons undergoing envelopment suggests that they were not apoptotic. Apoptosis is not the only mechanism of cell death. However, neuronal density in the cerebral cortex did not decline (Figure 5B), despite a higher than 20% rate of envelopment during the clinical stage that lasted 1 month (Figure 9A). Collectively, these findings argue that partially enveloped neurons remain viable. Aguzzi and coauthors suggested that the lack of neuronal death could be due to the relatively early endpoint imposed by animal welfare regulations (59). A 20% weight loss, used here as an endpoint, happens when animal’s motor impairment affects its ability to reach food and water in a cage. Previous studies have documented the survival of 22L-infected mice for up to 3 weeks after a 20% weight loss (60). It is unknown whether SSLOW-infected mice would experience any neuronal loss in the cerebral cortex upon approaching the actual endpoint.

The functional consequences of neuronal envelopment remain unclear. One hypothesis suggests that envelopment serves a neuroprotective role and is intended to be partial. If so, microglia might screen for stressed neurons before establishing contacts with them. Indeed, prior to the encircling stage, there is a decline in neuronal function, as evidenced by a drop in Grin1 signal. Activated microglia might survey neuronal health by partially encircling the neuronal surface. However, if neuronal-microglia interactions are purely protective, it is puzzling why the SSLOW strain, which has the shortest incubation time to the disease, shows the highest percentage of neuronal envelopment events. According to the alternative hypothesis, partial envelopment represents the initial step of neuronal engulfment by reactive microglia. If this is the case, it remains unclear why most encircling events were only partial and whether the engulfment is arrested or terminated prematurely. In SSLOW-infected mice, envelopment persisted at a rate of 20% or above for several weeks, while neuronal density did not decrease. This suggests that the low percentage of fully enveloped neurons may be due to a specific state of reactive microglia that cannot complete the process or a crosstalk between microglia and neurons (61), which remain viable while partially enveloped. Neurons might employ an unknown mechanism that resists microglial attempts at phagocytosis. The premature termination of full engulfment could result from overloading microglia with PrP^Sc^, which they cannot digest, potentially driving microglia to a senescent state. Consistent with this hypothesis, previous studies have shown that microglia acquire a senescent phenotype in cultures upon phagocytosing live neurons with tau aggregates (11, 12). Notably, the observation of cCasp3 in reactive microglia in this study aligns with previous work on Alzheimer’s and Parkinson’s diseases, where activation of caspase-3 in microglia was found to regulate the switch between proinflammatory activation and cell death (45, 46). Additionally, activation of caspase-3 has been shown to regulate cell proliferation (62). Overall, the current study reveals a unique pattern of body-to-body neuron-microglia cell interaction in the context of neurodegenerative diseases.

Together with CD18, CD11b constitutes complement receptor 3 (CR3). The CR3-dependent phagocytic pathway involves the complement factor C3b, which tags neurons and synapses, driving phagocytosis through interaction with CR3 (1, 2, 8). The CD11b-dependent pathway has been identified as responsible for the phagocytosis of neurons during development (37, 51). Furthermore, CR3-dependent mechanisms have been implicated in eliminating synapses and neurons in Alzheimer’s disease, frontotemporal dementia, and normal aging (8, 9, 63, 64). A substantial upregulation of *C3* and *Itgam*, the gene encoding CD11b, was observed in prion-infected mice and sCJD individuals (54, 65). However, the knockout of CD11b in the current study did not affect the engulfment of neurons by microglia, nor did it alter the incubation times to terminal disease. These findings suggest that a CD11b-independent pathway orchestrates the envelopment of neurons in prion diseases.

An upregulation of Gal3 with disease progression (Figure 9, F and G), along with an additional upregulation upon CD11b knockout, points to the Gal3-dependent pathway as a plausible mechanism responsible for neuronal envelopment (Figure 11, C and D). Gal3, released by activated myeloid cells, acts as an opsonin by binding to galactose residues of *N*-linked glycans lacking terminal sialic acid residues (43, 44).

It has been shown that during brain development, microglia can phagocytose viable nonapoptotic cells, including neural precursor cells (40, 66). Moreover, microglial attacks on viable nonapoptotic cells have also been suggested in neurodegenerative diseases (reviewed in ref. 7). In inherited retinal degeneration, microglia have been shown to phagocytose nonapoptotic photoreceptor cells (67). Surprisingly, injections of Aβ into the mouse brain were found to induce the phagocytosis of neurons by microglia (68).

Extensive body-to-body neuron-microglia cell interactions presented here reinstates the debate about the protective versus neurotoxic role of microglia. The uptake of PrP^Sc^ by microglia during the early stages of the disease, as shown here, supports the hypothesis of the protective role of microglia. Previously, ablation of microglia either before prion infection or during the early stages of the disease was found to accelerate disease progression, establishing their protective role (29–32). However, partial inhibition of microglia proliferation and reactivity at the late preclinical stage delayed disease onset and extended survival by 26 days (34). Moreover, therapeutic inhibition of microglia activation just before or after disease onset prolonged the survival of humanized mice infected with sCJD (35). These studies point to the transformation of the microglial role from predominantly positive during the early stage to a net negative during the late stages of the disease. We propose that this pivotal shift occurs at the late preclinical stage, where phagocytic uptake of PrP^Sc^ slows down and does not catch up with prion replication. Sustained phagocytic activity, initially a defensive response to prion infection, may eventually become detrimental due to the upregulation of phagocytic pathways. Indeed, substantial upregulation of phagocytic activity in reactive microglia associated with prion diseases has been documented (48–50). Moreover, phagocytic microglia did not discriminate between synaptosomes purified from prion-infected brains and normal synaptosomes as phagocytic substrates (49). Our current work suggests that the timing of the shift in phagocytic activity is crucial, emphasizing the need for a comprehensive understanding of the intricate interplay between microglial phagocytosis and the microglia-neuronal interaction.

## Methods

### Sex as a biological variable.

Our study examined male and female C57BL/6J mice and humans, and similar findings are reported for both sexes.

### Animals.

10% (w/v) SSLOW, RML, 22L, or ME7 brain homogenates (BH) for inoculations were prepared in PBS, pH 7.4, using glass/Teflon homogenizers attached to a cordless 12 V compact drill as previously described (69). SSLOW, RML, 22L, and ME7 brain-derived materials were inoculated as 1% or 10% BH via i.p. or i.c. route into C57BL/6J mice (Veterinary Resources, University of Maryland, Baltimore, Maryland, USA). Immediately before inoculation, each inoculum was further dispersed by 30 seconds indirect sonication at approximately 200 watts in a microplate horn of a sonicator (Qsonica). Each mouse received 20 μl of inoculum i.c. or 200 μl i.p. under 3% isoflurane anesthesia. Clinical signs included clasping hind legs, difficulty walking, abnormal gait, nesting problems, and weight loss. The animals were deemed symptomatic when at least 2 clinical signs were consistently observed. The mice were euthanized when they were unable to rear and/or lost 20% of their weight. CD11b^–/–^ mice with a global knockout of CD11b used in the current work were on the C57BL/6J background, and are described elsewhere (70). Control groups were age-matched noninoculated mice. For caspase-3 immunostaining positive control, mice were subjected to transient middle cerebral artery occlusion (MCAO) as previously described (71). 5XFAD mice were bred and housed as previously described (71). 10% (w/v) BH were prepared in RIPA Lysis Buffer (Millipore Sigma) as previously described (69). Analysis of gene expression by NanoString was performed as described before (72). The datasets from the 22L-infected and mock-inoculated control groups were also used to report microglia-neuronal colocalization in Sinha et al. (49).

### Elevated plus maze test and scoring of clinical signs of prion disease.

Mice were tested once per week starting at the preclinical stage. In each session, a mouse was placed at the center of an elevated plus maze (EPM) and given 5 minutes to explore the maze. All movements were captured using video recording, and total timing spent in open arms, closed arms, or the center of the maze was quantified using ANY-maze software (version 6.33). After the first training session, mice naturally acquire a strong preference for the closed arms, i.e., avoiding open arms during the sessions that follow the training session until the clinical onset of the disease. The clinical onset was defined at a time point when mice lose their preference for the closed arms.

### Antibodies.

Primary antibodies used for immunofluorescence, immunohistochemistry, and immunoblotting were as follows: rabbit polyclonal anti-IBA1 (013-27691, Fujifilm Wako Chemicals USA); goat polyclonal anti-IBA1 (NB100-1028, Novus); chicken polyclonal anti-MAP2 (NB300-213, Novus); mouse monoclonal anti-NeuN, clone A60 (MAB377, MilliporeSigma); rat monoclonal anti-CD68, clone FA-11 (MCA1957, Bio-Rad); mouse monoclonal anti-PV, clone PARV-19 (MAB1572, MilliporeSigma); rabbit polyclonal anti–caspase-3, active form (AB3623, MilliporeSigma); mouse monoclonal anti-prion protein, clone SAF-84 (189775, Cayman); rabbit monoclonal anti-prion protein, clone 3D17 (ZRB1268, MilliporeSigma); rabbit monoclonal anti-GFAP, clone D1F4Q (12389, Cell Signaling Technology); chicken polyclonal anti-GFAP (AB5541, MilliporeSigma); mouse monoclonal anti–β-amyloid, clone 6E10 (803004, BioLegend); rat monoclonal anti-LAMP1, clone 1D4B (121601, BioLegend); rabbit polyclonal anti-CD11b (ab128797, Abcam); mouse monoclonal anti-tubulin β3, clone TUJ1 (801213, BioLegend); rat monoclonal anti-Gal3, clone M3/38 (sc-23938, Santa Cruz Biotechnology Inc.); mouse monoclonal anti–β-actin, clone AC-15 (A5441, Sigma-Aldrich); rabbit polyclonal Grin1 (27676-1-AP, Proteintech); and rabbit monoclonal cathepsin D (69854, Cell Signaling Technology). The secondary antibodies for immunofluorescence were Alexa Fluor 488, 546, and 647 labeled (Thermo Fisher Scientific).

### Immunofluorescence and DAB staining of mouse brains.

Formalin-fixed brains (sagittal or coronal 3 mm slices) were treated for 1 hour in 96% formic acid before being embedded in paraffin using standard procedures; 4 μm sections produced with Leica RM2235 microtome (Leica Biosystems) were mounted on Superfrost Plus Microscope slides (22-037-246, Fisher Scientific) and processed for immunohistochemistry according to standard protocols. To expose epitopes, slides were subjected to 20 minutes of hydrated autoclaving at 121°C in citrate buffer, pH6.0, antigen retriever (C9999, Sigma-Aldrich). For the detection of disease-associated PrP, an additional 3 minutes treatment in concentrated formic acid was applied.

For immunofluorescence, an Autofluorescence Eliminator Reagent (Sigma-Aldrich) and Signal Enhancer (Thermo Fisher) were used on slides according to the original protocols to reduce background fluorescence. Epifluorescent images were collected using an inverted microscope Nikon Eclipse TE2000-U (Nikon Instruments Inc.) equipped with an illumination system X-cite 120 (EXFO Photonics Solutions Inc.) and a cooled 12-bit CoolSnap HQ CCD camera (Photometrics). Images were processed using ImageJ software (2.14.0, NIH). Full-brain tile scan images were acquired and stitched using Leica MICA widefield microscope with the ×20 dry objective. The 365 nm, 470 nm, and 555 nm LEDs were used at a resolution of 2432px × 2032px per tile. The stitched images were then processed with Leica’s Instant Computational Clearing (ICC). 3D movies in Supplemental Figure 4 were produced with 3D images acquired using Leica MICA widefield microscope with the ×63/1.20 water immersion objective. The 365 nm, 470 nm, and 555 nm LEDs were used at a resolution of 2432px × 2032px for 45 slices and a *Z*-stack size of 6–7 μm.

For detection with DAB, horseradish peroxidase–labeled secondary antibodies and DAB Quanto chromogen and substrate (VWR, Radnor) were used. The images were acquired with Boreal 2 light microscope equipped with digital camera and processed with Motic Image Plus 3.0 software (Boreal Science, VWR).

### Immunohistochemistry of sCJD brains.

Immunohistochemistry of 57 sCJD brains, including 42 from the 3 most common disease subtypes (MM1, VV2, and MV2K) was performed as described before (73). Briefly, 7 mm sections from formalin-fixed and paraffin-embedded tissue blocks were stained using a monoclonal antibody against the b-chain of human HLA-DR, -DQ, and -DP (clone CR3/43, 1:400; Agilent Dako).Antigen retrieval was achieved by microwaving the sections in sodium citrate buffer (0.01 M, pH 6.0, 15 minutes). Slides were then loaded into an Aperio ScanScope XT (Aperio, Leica Biosystems) and scanned at ×20 magnification (1 mm/pixel) via the semi-automated method. Slides were checked for image quality using an Aperio “quality factor >90” and visual inspection and annotated using the ImageScope, version 12, software (Leica Biosystems).

### TUNEL assay.

Click-IT Plus TUNEL Assay (C10617, Thermo Fisher) was performed on brain sections prepared without formic acid treatment. The TUNEL assay protocol recommended by the manufacturer was adapted for staining of free-floating sections within a 24-well plate. Following the TUNEL reaction, brain sections were immunostained for IBA1 to allow the visualization of microglia.

### Confocal microscopy and 3D image reconstruction.

Confocal images were acquired using a Leica TCS SP8 microscope using the ×40/1.30 or ×63/1.40 oil immersion objective lenses with laser lines 405, 488, 552, and 638 when needed. Image resolution was 1024 × 1024 or 2048 × 2048 pixels at a 400 Hz scan speed. *Z*-stack thickness ranged from 6.2 to 10.45 μm taken at the system-optimized number of steps. Images were processed using the LAS X, version 1.4.5.27713, and ImageJ.

### Quantification of envelopment and neuronal count.

To estimate the percentage of neurons enveloped by microglia, brains were coimmunostained for MAP2 and IBA1, and multiple images from cortices of each brain were collected under a ×60 objective. Using ImageJ software, images obtained from green and red channels were subjected to an automated threshold, and for each MAP2^+^ neuron, an area of overlap between signals from MAP and IBA1 channels was recorded. Based on the calculations for control brains, 20-pixel overlap was chosen as a cut-off for the detection of envelopment events. Neurons were counted as undergoing envelopment, if they had over 20 pixels of MAP2 signal overlapped by IBA1 signal. The total number of identified MAP2^+^ cells was recorded as a neuronal count.

To estimate the percentage of PV^+^ neurons undergoing envelopment, brains were coimmunostained with anti-PV and anti-IBA1 antibodies, and multiple images from cortices of each brain were collected under a ×20 objective. Images from PV and IBA1 channels were thresholded, converted to binary, and PV^+^ neurons were identified as regions of interest (ROIs) using the Analyze Particles function of ImageJ. The same ROIs were applied to corresponding images from the IBA1 channel, and the area of IBA1 signal overlap with each PV^+^ neuron was recorded. An area of 20 pxls or more was counted as envelopment.

To assess the frequency of complete engulfment events, the images were collected from cortices of brains coimmunostained for NeuN and IBA1 under a ×20 objective. The neurons were identified using the Analyze Particles function of ImageJ software as above. The percentage of NeuN signal area covered by IBA1 signal was recorded for each neuron.

### Quantification of neuronal and microglial density in the pyramidal layer of hippocampus.

Brain tissues coimmunostained for IBA1 and NeuN were imaged under a ×20 objective. For each image, the mean intensity of IBA1 and NeuN was measured in 1 to 3 rectangles (60 × 200 pixels) encompassing pyramidal neurons in the CA1 region of the hippocampus.

### Quantification of cCasp3^+^ cells.

To compare the number of cCasp3^+^ neurons and microglia cells, 2 brains of terminal SSLOW-infected C57BL/6J mice and 2 brains of normally aged (607, 740 days old) C57BL/6J mice were coimmunostained for cCasp3 and NeuN or IBA1, and images from the cortices of each brain were collected under a ×20 objective. The images from each channel were subjected to a set threshold, converted to binary, and neurons or microglia cells were identified as ROIs using the Analyze Particles function of ImageJ software. The same ROIs were applied to the corresponding cCasp3 channel, and the presence of the cCasp3 signal in each identified cell was measured.

### Colocalization of PrP signal with different cell types.

Brains of terminal SSLOW-infected mice were coimmunostained for PrP (3D17) and cell-type markers MAP2, GFAP, and IBA1. The images from each channel were subjected to a set threshold, and an area of overlap between PrP signal and MAP2, GFAP, or IBA1 was detected using the Image Calculator function of ImageJ. Total 3D17 signal intensity and 3D17 signal intensity in the areas of overlap were measured.

### Quantification of PrP^Sc+^ microglia.

Brains were coimmunostained with anti-PrP 3D17 and IBA1 antibodies. The images from each channel collected under a ×60 objective were subjected to a set threshold, converted to binary, and microglia cells were identified as ROIs using the Analyze Particles function of ImageJ. The same ROIs were applied to the corresponding 3D17 channel, and the presence of the PrP^Sc^ signal in each identified microglia cell was measured. The number of microglia cells identified in each image was also recorded.

### Quantification of cathepsin D in microglia.

Images taken with a ×20 objective were subjected to automatic subtraction of background (50 pixels rolling ball radius) and 8-bit conversion using ImageJ software. Cathepsin D and IBA1 channels were thresholded and made binary; then image calculator AND operation was applied to a select pixel with both cathepsin D and IBA1 signals. Integrated density of the resulting area was calculated on the original 8-bit cathepsin D image representing the cathepsin D associated with microglia.

### Quantification of LAMP in microglia and Grin1 in neurons.

IBA and LAMP1 images collected with confocal microscopy were merged, and background was subtracted. Then single microglial cells were selected, opened as separate images, and split into channels and thresholded using the Yen method (74). The area and mean gray value of each microglia cell were measured, and the area of LAMP1 inside microglia was determined using the Image Calculator function with the AND operation. Mean gray value of LAMP1 inside microglia was determined by overlaying LAMP1 binary selection from previous step onto the original image and measuring intensity.

IBA1, Grin1, and NeuN images were collected with confocal microscopy using the same acquisition settings. NeuN and Grin1 were merged and ROIs were drawn around neurons in the field of view. The mean intensity of Grin1 in each ROI was measured using ImageJ.

### Statistics.

Statistical analyses and plotting of the data were performed using GraphPad Prism software, versions 8.4.2–10.1.1 for Windows (GraphPad Software), or Excel 2016–version 2302, as detailed in figure legends.

### Study approval.

The study was carried out in strict accordance with the recommendations in the *Guide for the Care and Use of Laboratory Animals* of the National Institutes of Health (National Academies Press, 2011). The animal protocol was approved by the Institutional Animal Care and Use Committee of the University of Maryland, Baltimore (assurance number A32000-01; permit number 0215002). Human brain tissues have been collected under the Italian National Surveillance program for CJD and related disorders, and their use for research was approved with written informed consent of patients during life or their next of kin after death.

### Data availability.

All data associated with this study are presented in the paper or in the Supplemental Methods. Values for all data points in graphs are reported in the Supporting Data Values file.

## Author contributions

NM and IVB conceived the project. NM, TS, and OB developed methodology. NM, TS, OB, and OM performed formal analysis. NM, TS, OM, NPP, KM, SB, and PP performed experiments. LZ and PP provided resources. IVB wrote the original draft. NM, TS, OB, OM, NPP, KM, SB, LZ, and PP reviewed and edited the manuscript. IVB supervised the project. NM and IVB performed project administration. IVB and LZ acquired funding.

## Supplementary Material

Supplemental data

Unedited blot and gel images

Supplemental video 1

Supplemental video 2

Supplemental video 3

Supplemental video 4

Supplemental video 5

Supporting data values

## Version 1 10/03/2024

In-Press Preview

## Version 2 12/02/2024

Electronic publication
