# Supplementary material for: Reactive microglia partially envelop viable neurons in prion diseases
Source: J Clin Invest. 2024 Oct 3;134(23):e181169. doi: 10.1172/JCI181169 (PMC11601909; doi:10.1172/JCI181169)
Supplement: Supplemental data [file jci-134-181169-s035.pdf]

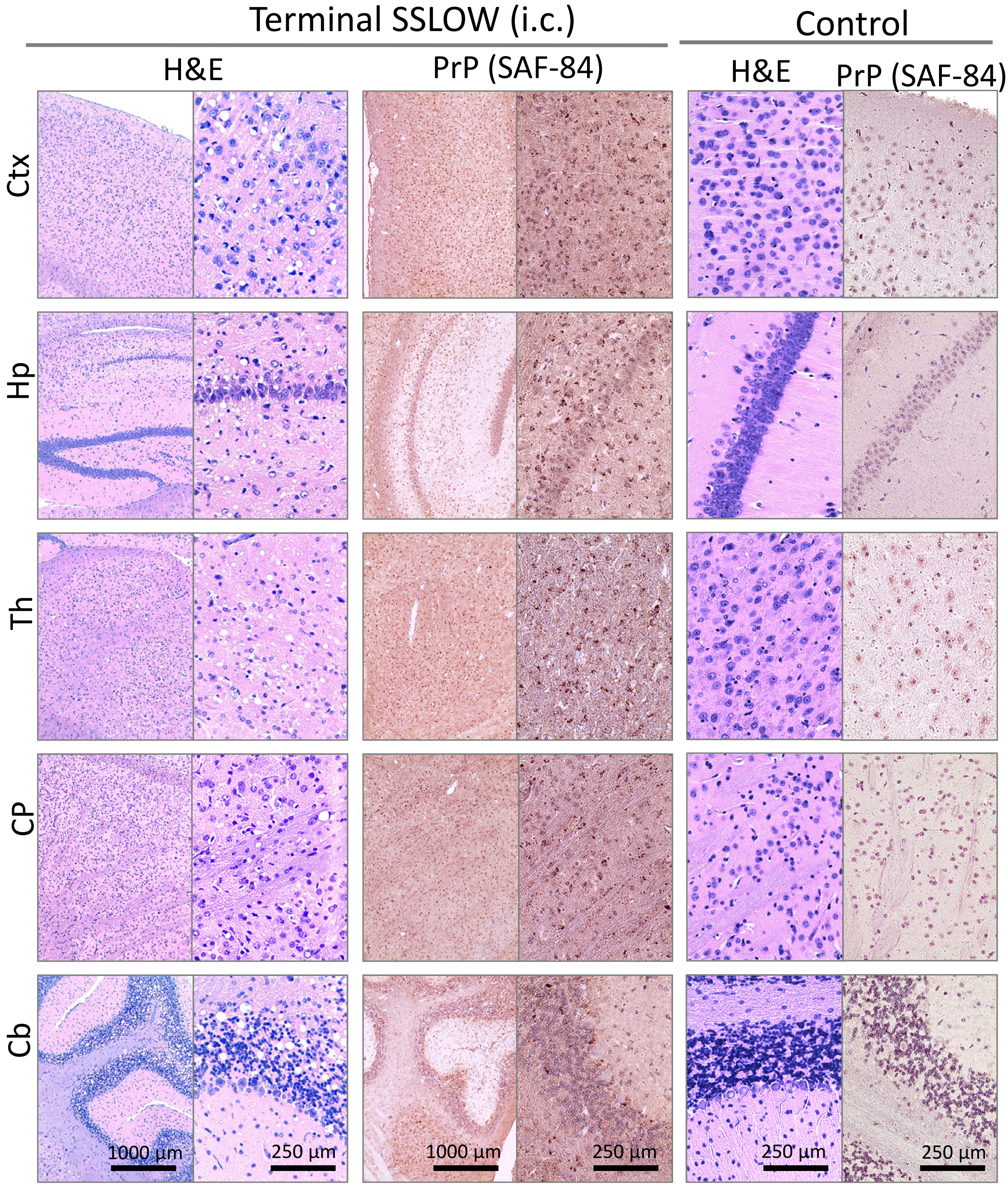

Supplemental Figure 1. Vacuolation and PrP^{Sc} deposition in C57Bl/6J mice infected with SSLOW. C57Bl/6J mice challenged with SSLOW via i.c. route or non-infected age-matched control mice were analyzed for spongiform vacuolation using staining with hematoxylin and eosin, and PrP^{Sc} deposition using staining with anti-PrP SAF-84 antibody. Ctx - cortex, Hp - hippocampus, Th - thalamus, CP - caudate putamen, Cb - cerebellum.

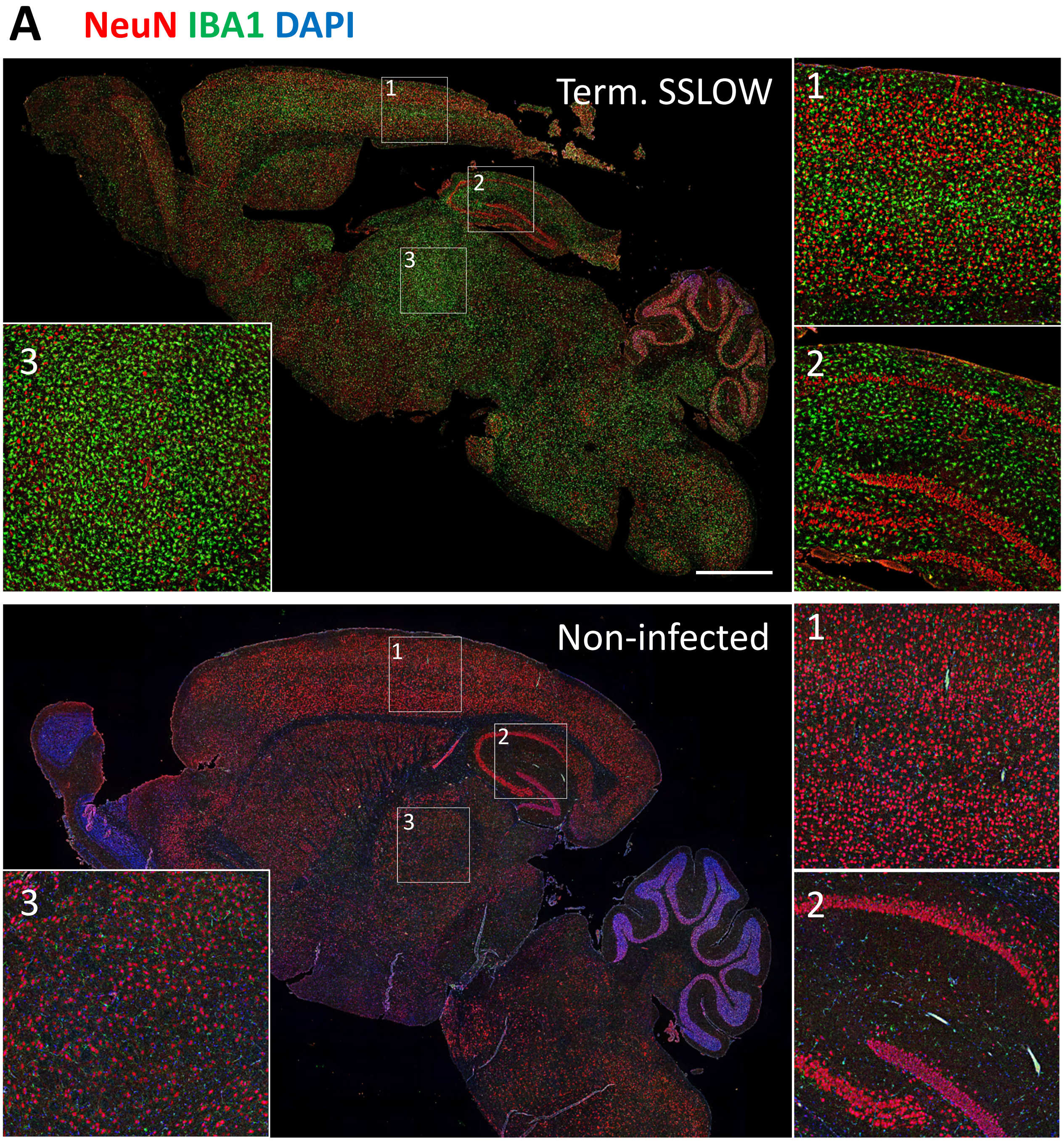

Supplemental Figure 2A. Full section scans of terminal C57Bl/6J mice challenged with SSLOW via i.c. route and non-infected age-matched control C57Bl/6J mice. Brains immunostained for neurons (NeuN, red) and microglia (IBA1, green). Insets show magnified images of: 1 – cortex, 2 – hippocampus, 3 – thalamus. The scans were performed using MICA microscope. Scale bar = 1 mm.

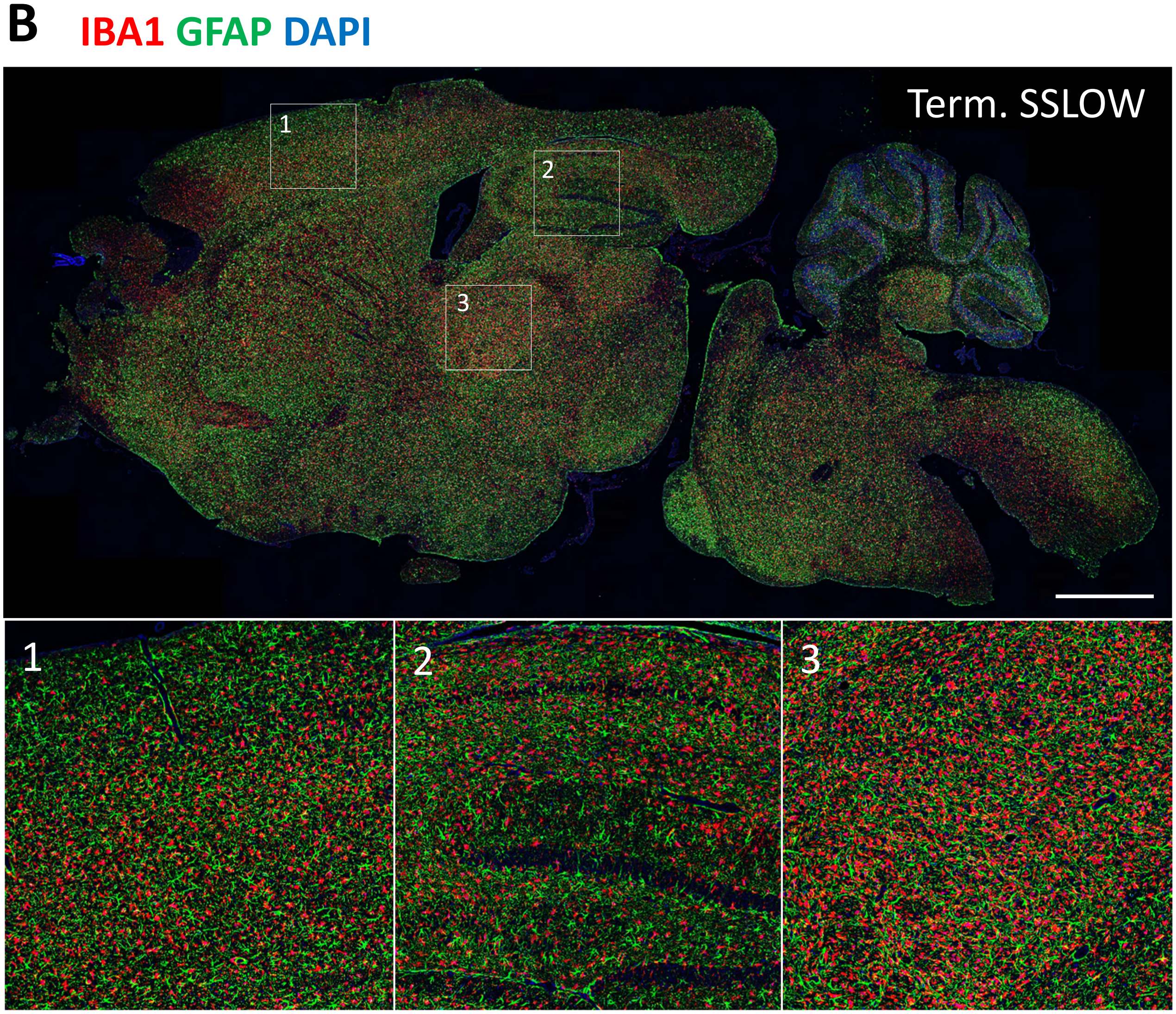

Supplemental Figure 2B. Full section scans of terminal C57Bl/6J mice challenged with SSLOW via i.c. route. Brains immunostained for astrocytes (GFAP, green) and microglia (IBA1, red). Insets show magnified images of: 1 – cortex, 2 – hippocampus, 3 – thalamus. The scans were performed using MICA microscope. Scale bar = 1 mm.

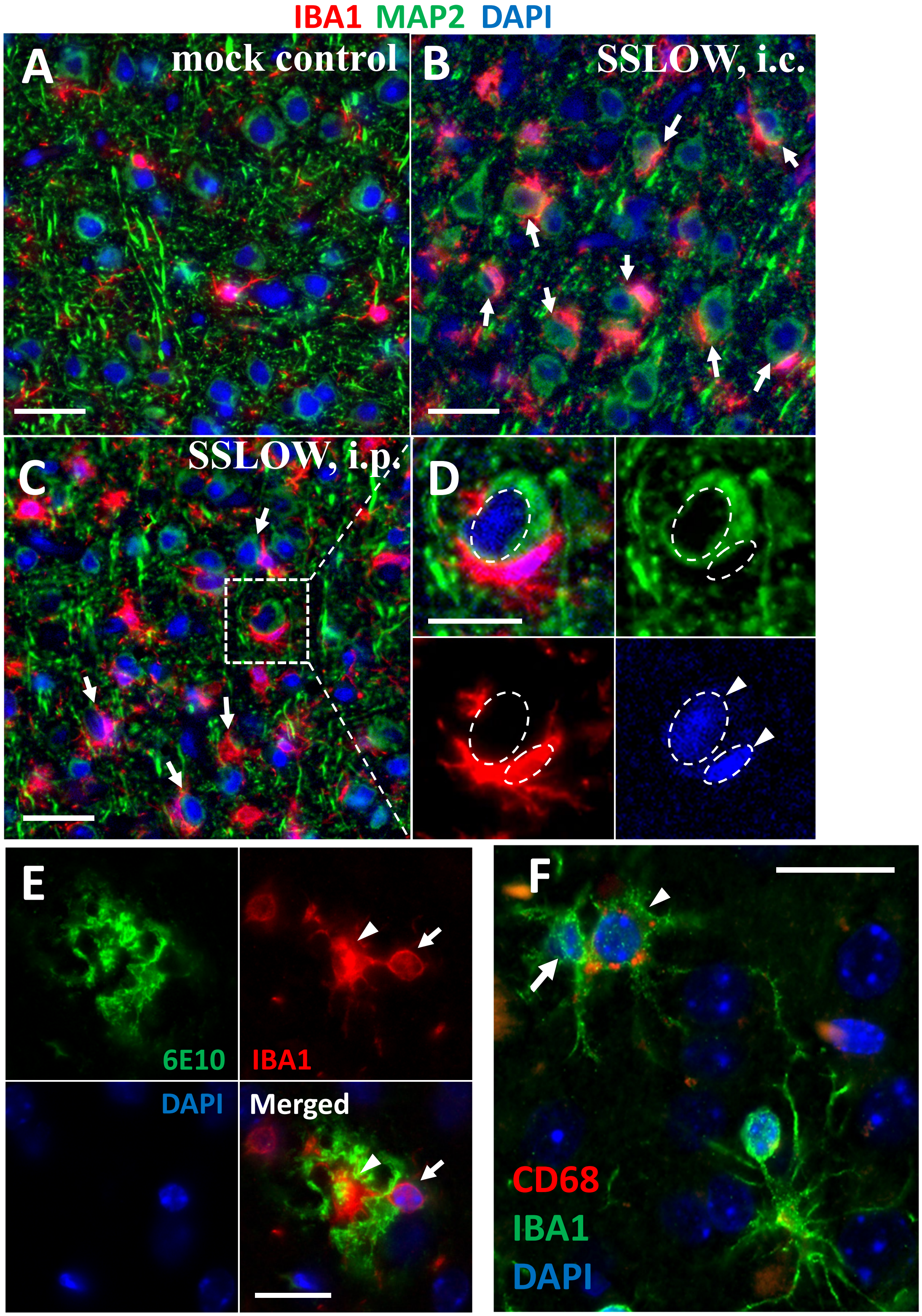

Supplemental Figure 3. Reactive microglia in prion-infected mice and 5XFAD mice. (A-D) Co-immunostaining of terminally ill C57Bl/6J mice infected with SSLOW via i.c. (**B**) or i.p. routes (**C**,**D**), and mock controls (**A**) using anti-IBA1 (a marker of microglia) and anti-MAP2 (a neuronal marker) antibodies. Arrows point at partially enveloped neurons. (**D**) Enlarged image of envelopment showing cup-shaped microglial soma. Arrowheads and dashed circles mark neuronal and microglial nuclei located in close proximity. (**E**,**F**) Co-immunostaining of 10-month-old 5XFAD mice using anti-IBA1 and 6E10 antibodies that stain Aβ amyloid plaques (**E**), or anti-IBA1 and anti-CD68 antibodies (**F**). In 5XFADs, microglia form pseudopodia with phagocytic cups shown by arrowheads. The arrows point at microglia soma. Scale bars = 20 μm in **A-C**,**F**, and 10 μm in **D** and **E**.

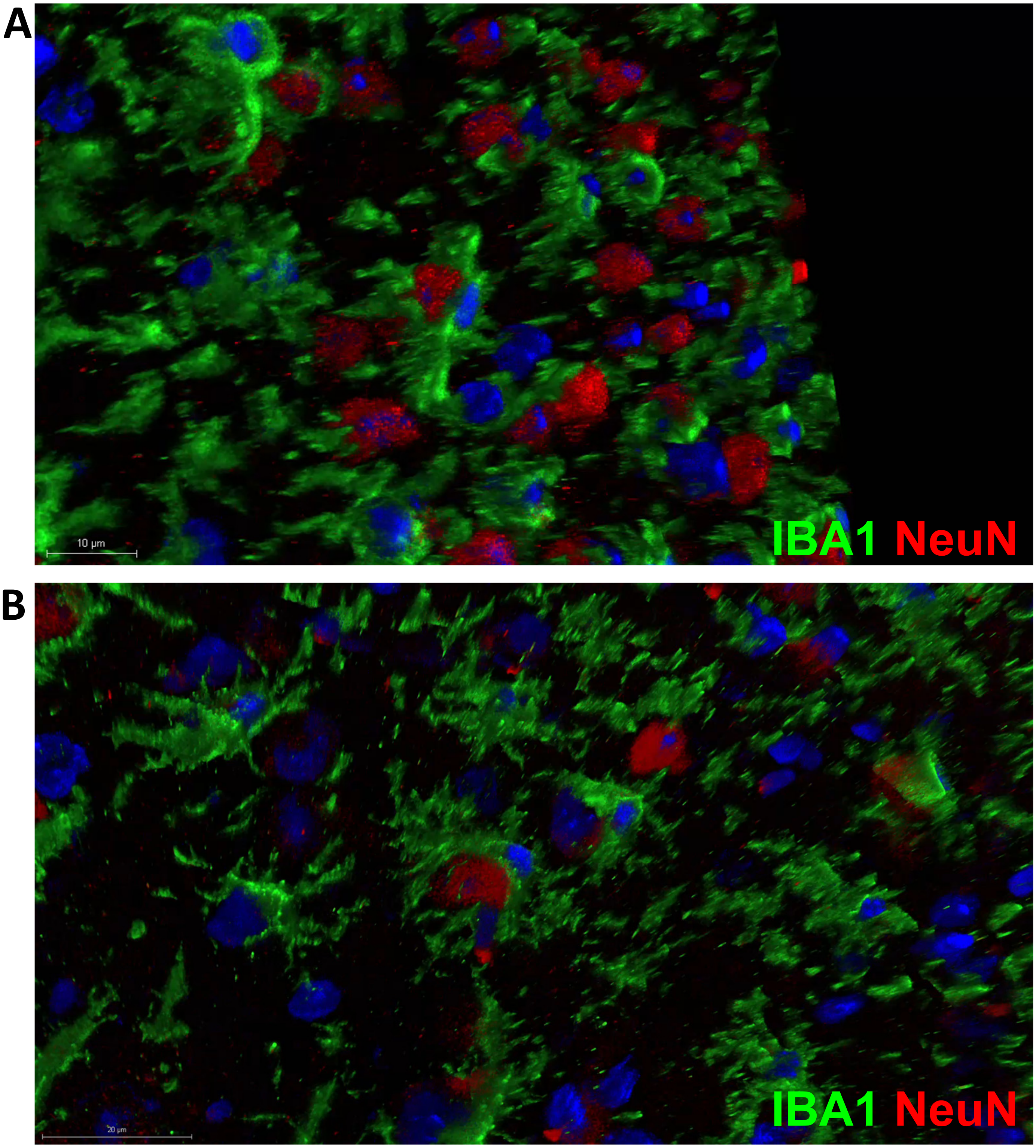

Supplemental Figure 4. MICA microscopy Z-stack videos (A, B) showing envelopment of neurons by reactive microglia. SSLOW-infected C57Bl/6J mouse brains collected at the terminal stage of the disease and coimmunostained using anti-IBA1 (green) and anti-NeuN (red) antibodies.

<u>Corresponding video is deposited under the file name "Figure S4A video and Figure S4B video"</u>

A

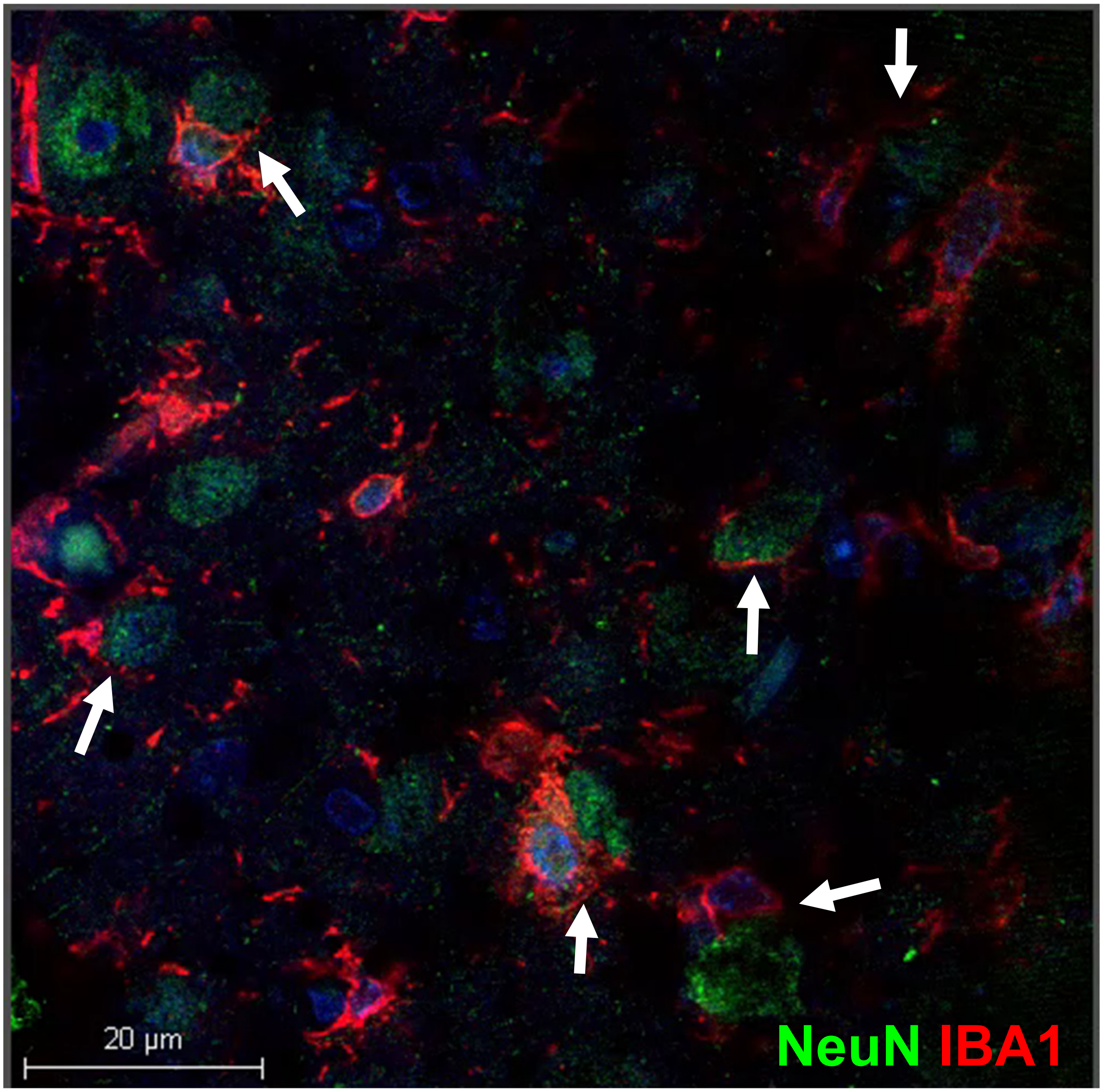

Supplemental Figure 5. Confocal microscopy Z-stack videos (A, B) showing envelopment of neurons by reactive microglia. SSLOW-infected C57Bl/6J mouse brains collected at the terminal stage of the disease and coimmunostained using anti-IBA1 and anti-NeuN antibodies. Arrows point at microglia that envelop neurons.

<u>Corresponding video is deposited under the file name "Figure S5A video"</u>

B

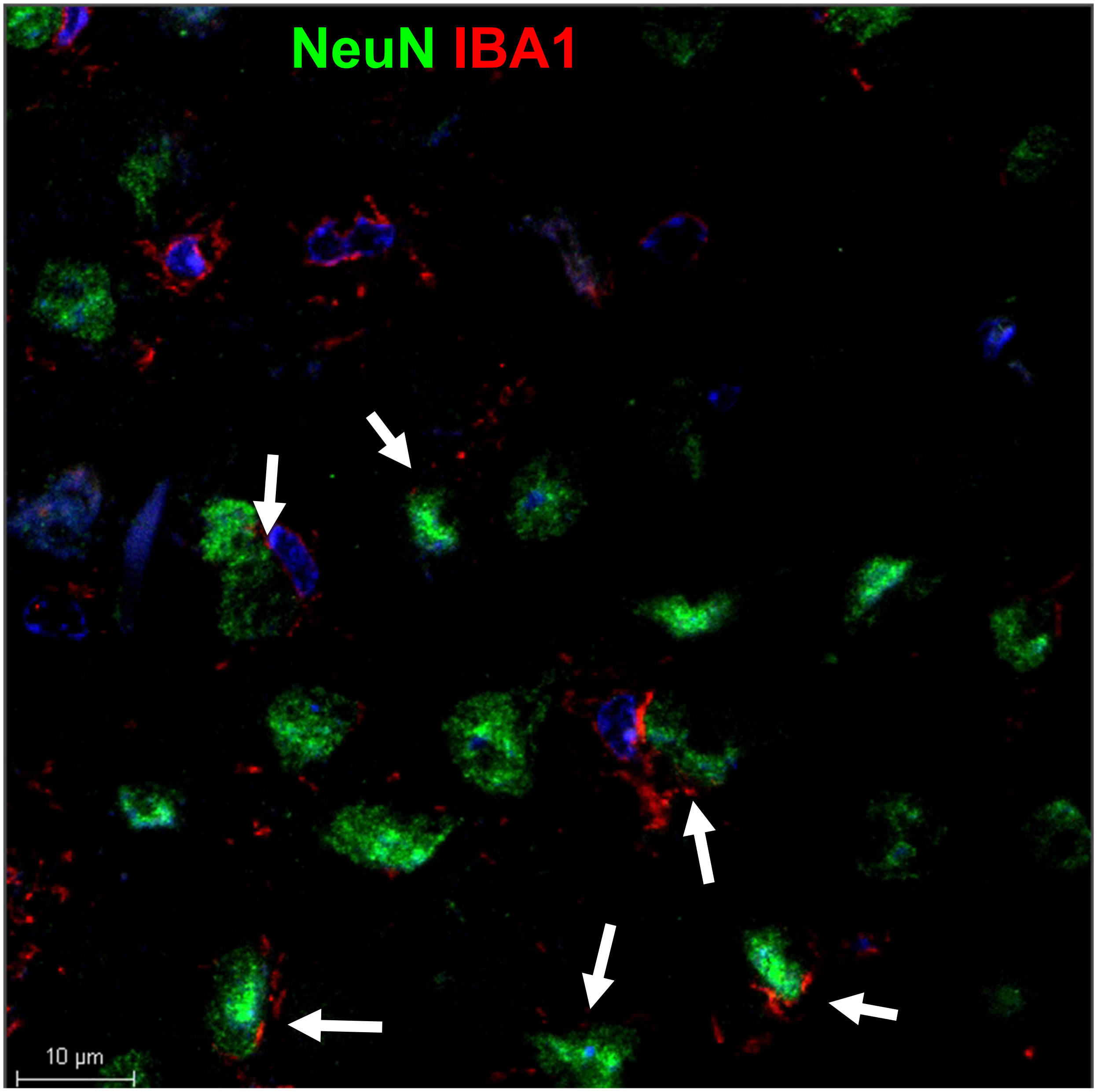

Supplemental Figure 5B.

<u>Corresponding video is deposited under the file name "Figure S5B video"</u>

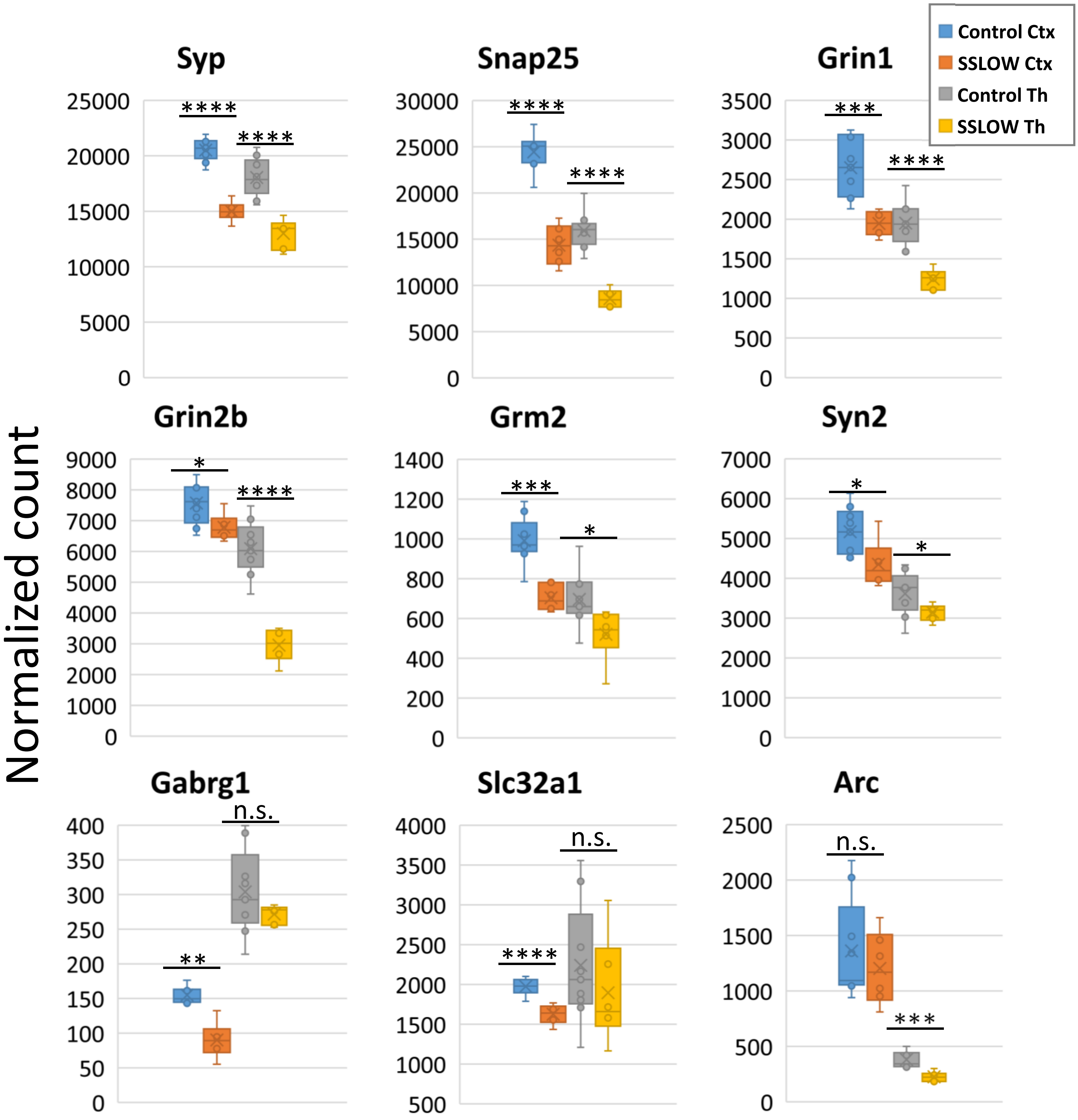

Supplemental Figure 6. Downregulation of genes responsible for neuronal function in prion disease. Normalized expression of genes responsible for neuronal function in C57Bl/6J mice infected with SSLOW via i.p. route or age-matched control animals analyzed by NanoString. Data are presented as box-and-whisker plots, where the midline denotes the median, the x represents the mean, and the ends of the box plot denote the 25th and 75th percentiles. N=9 for controls, N=6 for SSLOW-infected mice. **** $p < 0.0001$; *** $p < 0.001$; ** $p < 0.01$; * $p < 0.05$ by unpaired Student's t-test.

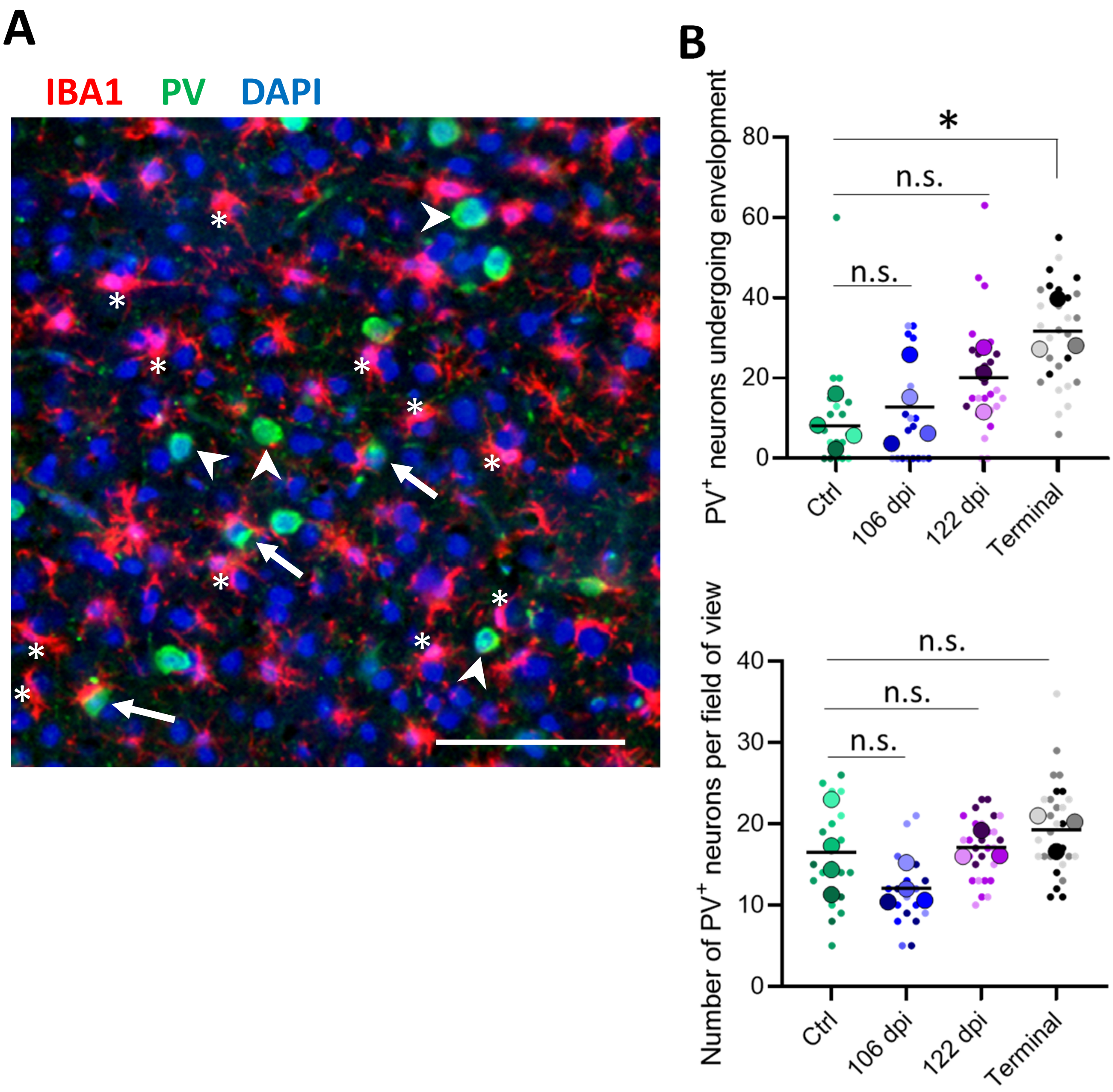

Supplemental Figure 7. Envelopment does not selectively target PV$^+$ neurons. (A) Coimmunostaining of cortexes of terminally ill C57Bl/6J mice infected i.p. with SSLOW, using anti IBA1 and anti-PV antibodies. Arrows indicate examples of partially enveloped PV$^+$ neurons; not enveloped PV$^+$ neurons are marked by arrowheads. Examples of microglia enveloping PV$^-$ cells are marked by asterisks. (B) The percentage of PV$^+$ neurons undergoing envelopment (upper plot) and number of PV$^+$ neurons (lower plot) at the preclinical stage (106 dpi), clinical onset (122 dpi) and terminal stage of the disease (157-166 dpi) in the cerebral cortex of mice inoculated i.p. with SSLOW. Colors represent different brains. Dots represent individual values. Average values for each brain are shown as circles, n=3-4 individual animals for each time point. Means are marked by black lines. * $p<0.05$, n.s. – non-significant, by Brown-Forsythe and Welch ANOVA followed by Dunnett's T3 multiple comparison tests. Scale bar = 50 µm.

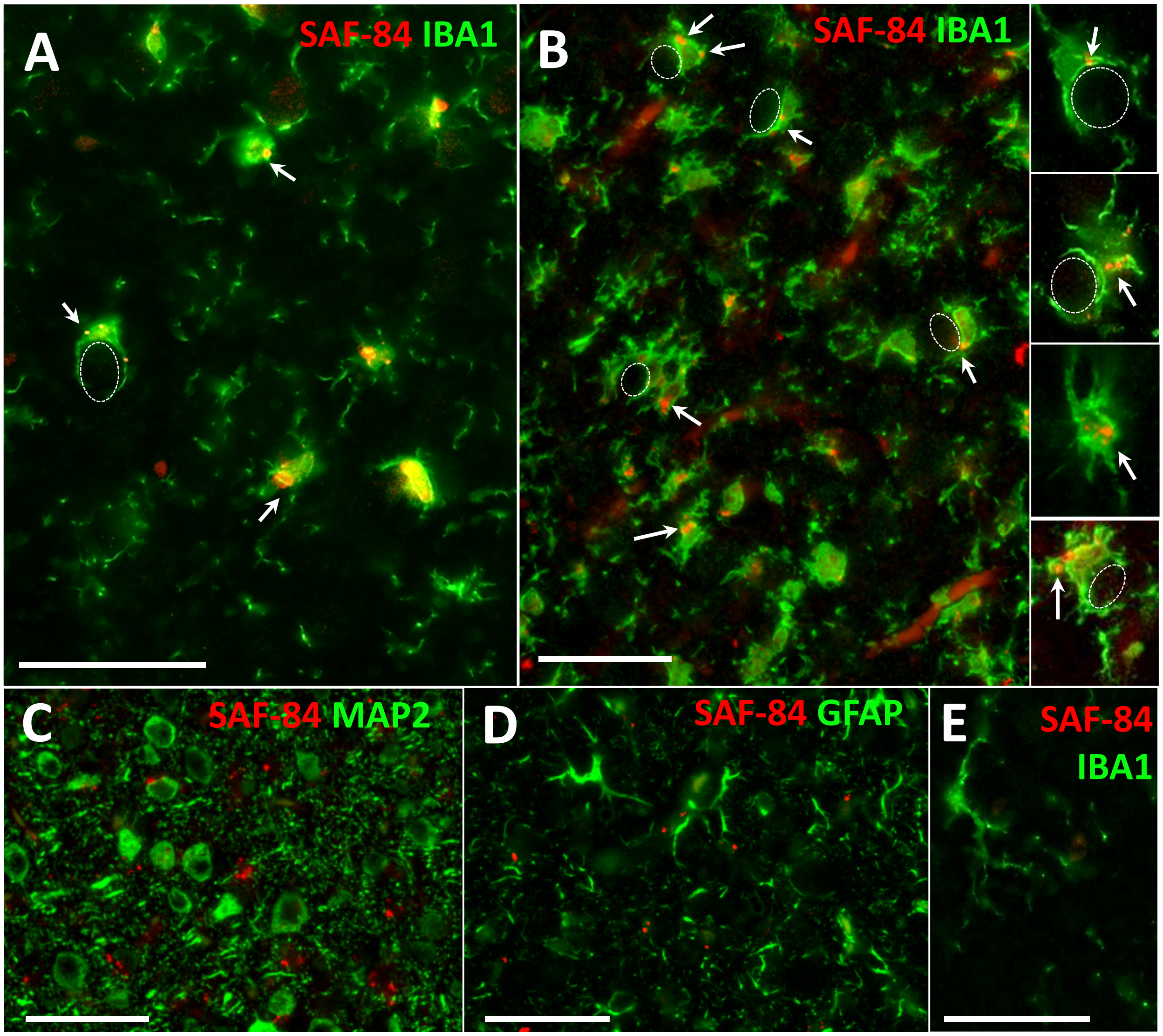

Supplemental Figure 8. PrP^{Sc} colocalizes with reactive microglia. (A-E) Coimmunostaining of SSLOW-infected C57Bl/6J mouse brains using anti-PrP (SAF-84) and anti-IBA1 antibodies at the preclinical (A) and terminal stages of the disease (B), or SAF-84 and anti-MAP2 (C) or anti-GFAP antibodies (D) at the terminal stage of the disease. Circles show neuronal envelopment. (E) Co-immunostaining of non-infected, age-matched C57Bl/6J mouse brains using SAF-84 and anti-IBA1 antibodies. Scale bar = 25 μm.

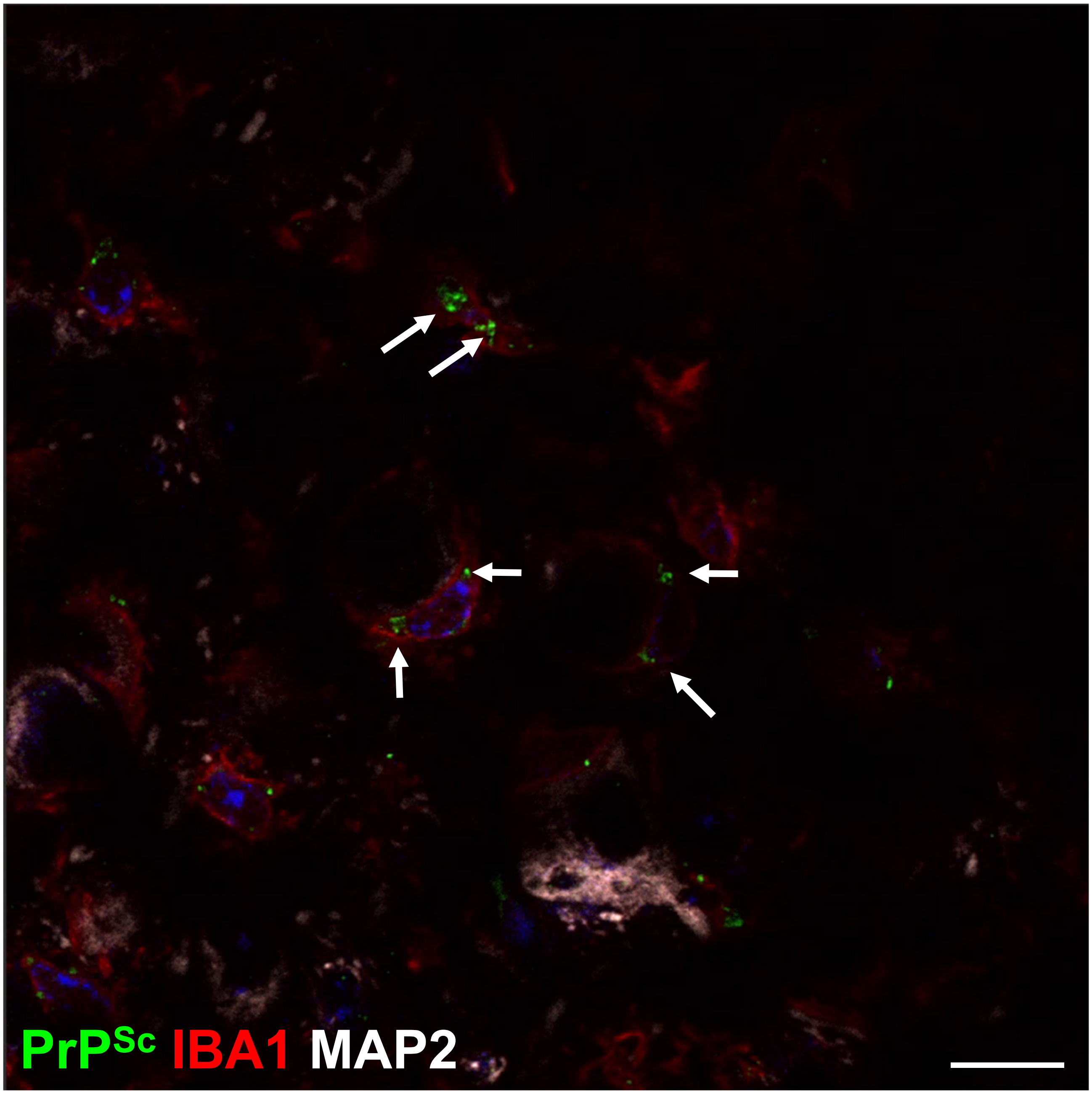

Supplemental Figure 9. Confocal microscopy Z-stack video showing envelopment of neurons by PrP^{Sc}-positive reactive microglia. SSLOW-infected C57Bl/6J mouse brains collected at the terminal stage of the disease and coimmunostained using anti-PrP (SAF-84), anti-IBA1 and anti-MAP2 antibodies. Arrows point at intracellular PrP^{Sc} deposits in microglia enveloping neurons. Scale bar = 10 µm.

Corresponding video is deposited under the file name "Figure S9 video"

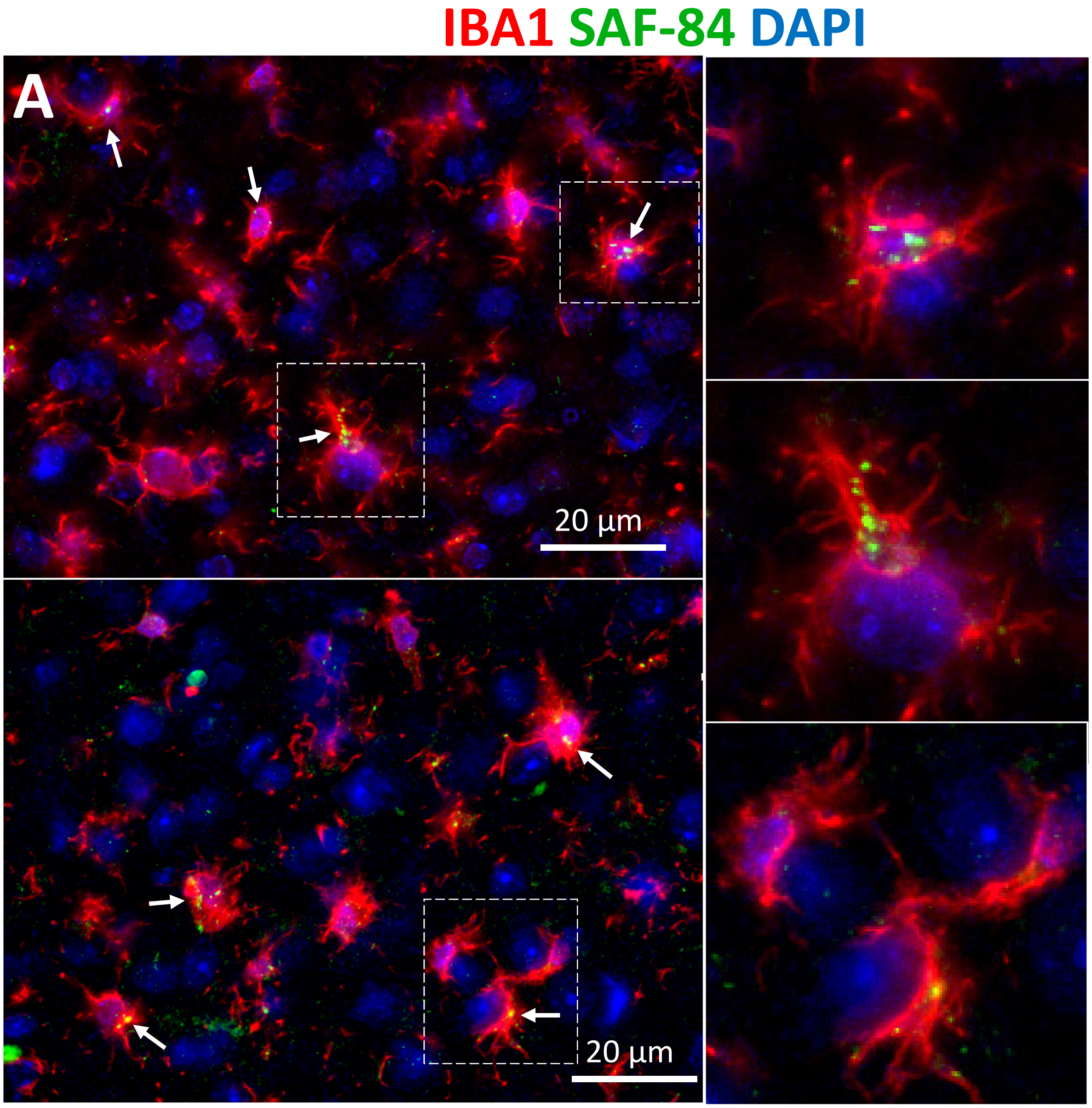

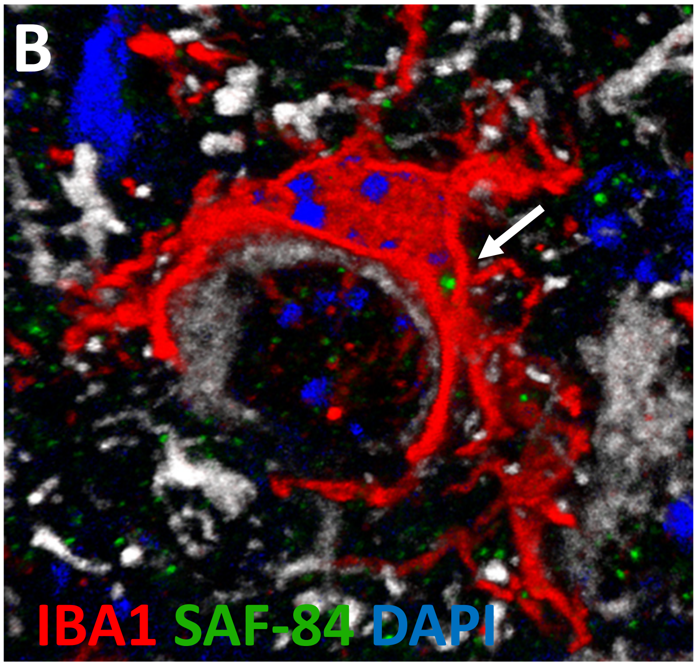

Supplemental Figure 10. Granular PrP^{Sc} colocalizes with reactive microglia in 22L-infected animals. (**A**) Representative images of PrP^{Sc} colocalized with IBA1+ cells in C57Bl/6J mice infected with 22L via i.c. route at terminal stages of the disease. (**B**) 3D reconstruction of confocal microscopy images of PrP^{Sc}+ positive microglia enveloping a neuron. Brain sections were stained with anti-PrP antibody SAF-84 (green) and anti-IBA1 antibody (red). Arrows indicate examples of PrP^{Sc}+ microglia. Scale bar = 20 µm.

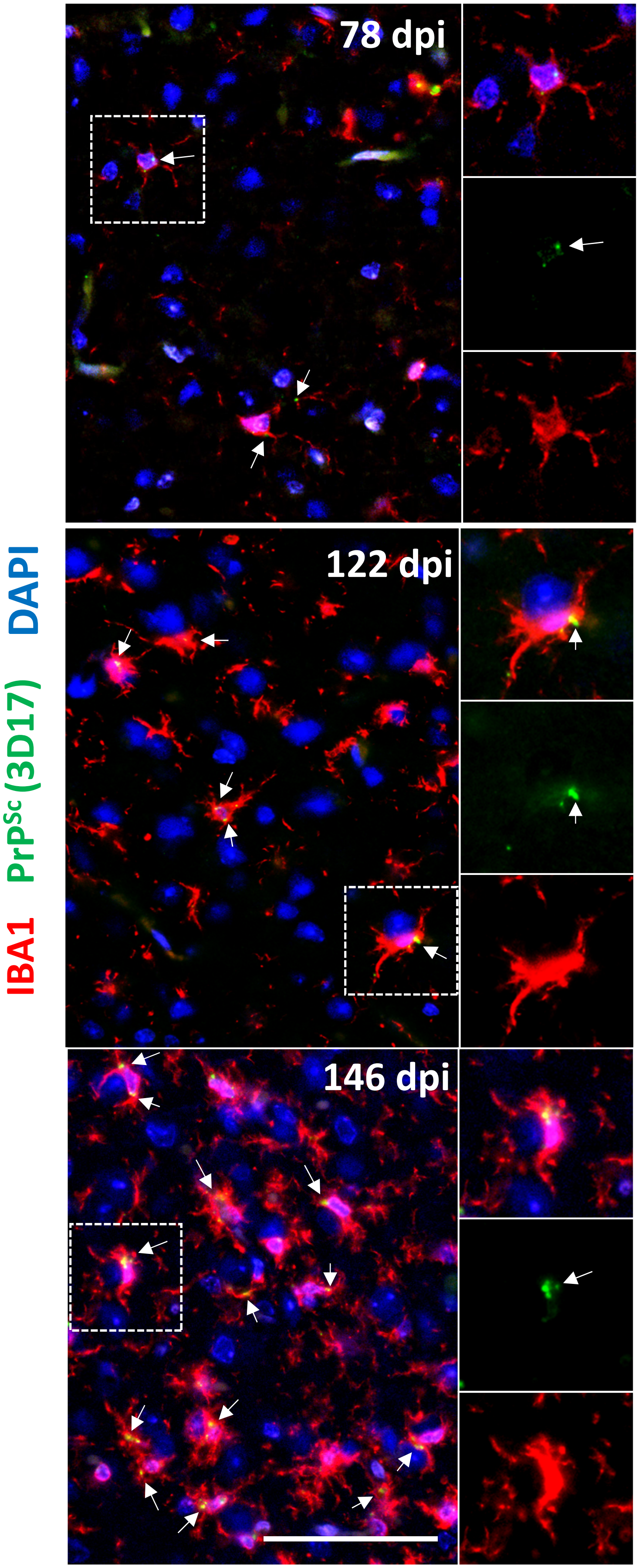

Supplemental Figure 11. Granular PrP^{Sc} colocalizes with reactive microglia in SSLOW-infected animals. Representative images of PrP^{Sc} colocalized with IBA1+ cells in C57Bl/6J mice infected with SSLOW via i.p. route at the preclinical (78 dpi), onset of symptoms (122 dpi), and clinical stages of the disease (146 dpi). Brain sections were stained with anti-PrP antibody 3D17 and anti-IBA1 antibody. Arrows indicate examples of PrP^{Sc}+ microglia. Scale bar = 50 µm.
