# Supplementary material for: Reactive microglia partially envelop viable neurons in prion diseases
Source: J Clin Invest. 2024 Oct 3;134(23):e181169. doi: 10.1172/JCI181169 (PMC11601909; doi:10.1172/JCI181169)
Supplement: Unedited blot and gel images [file jci-134-181169-s036.pdf]

Figure 9B

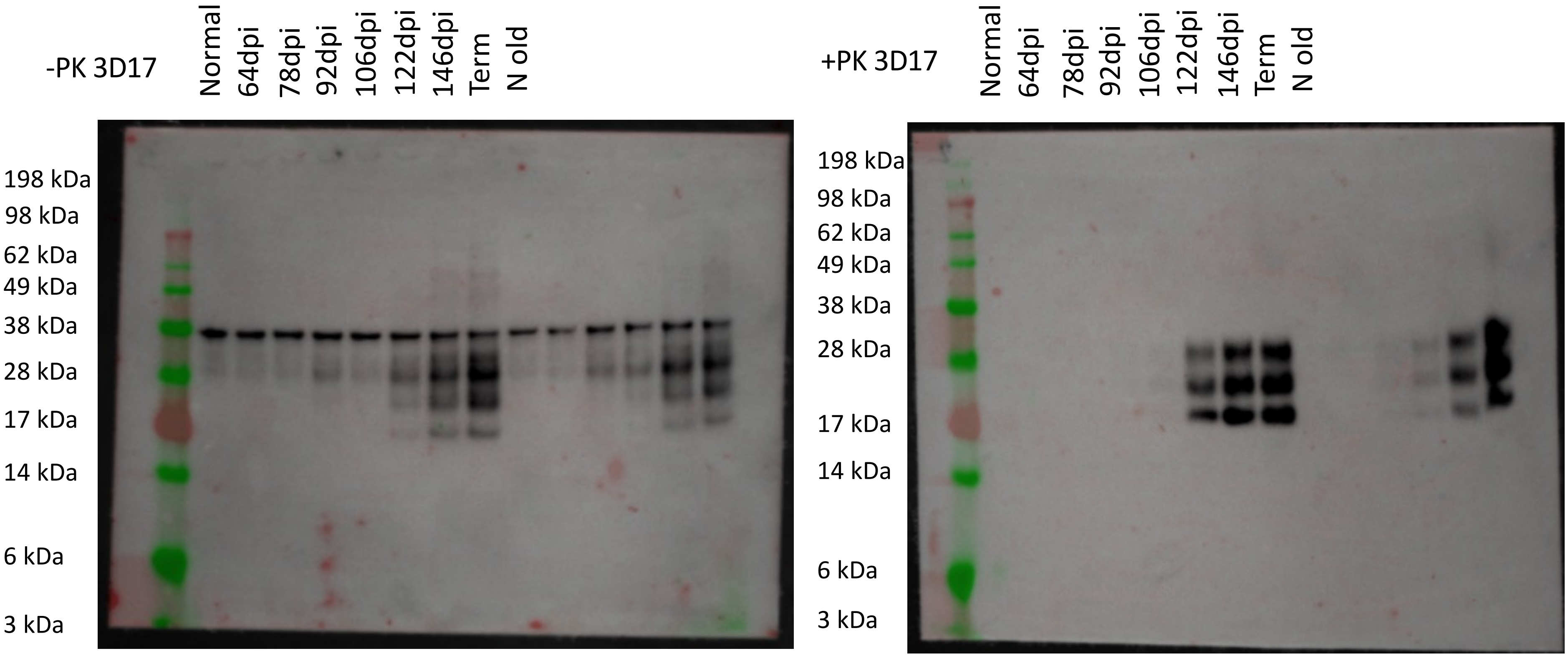

-PK 3D17
Normal
64dpi
78dpi
92dpi
106dpi
122dpi
146dpi
Term
N old
198 kDa
98 kDa
62 kDa
49 kDa
38 kDa
28 kDa
17 kDa
14 kDa
6 kDa
3 kDa
+PK 3D17
Normal
64dpi
78dpi
92dpi
106dpi
122dpi
146dpi
Term
N old
198 kDa
98 kDa
62 kDa
49 kDa
38 kDa
28 kDa
17 kDa
14 kDa
6 kDa
3 kDa

Figure 9B

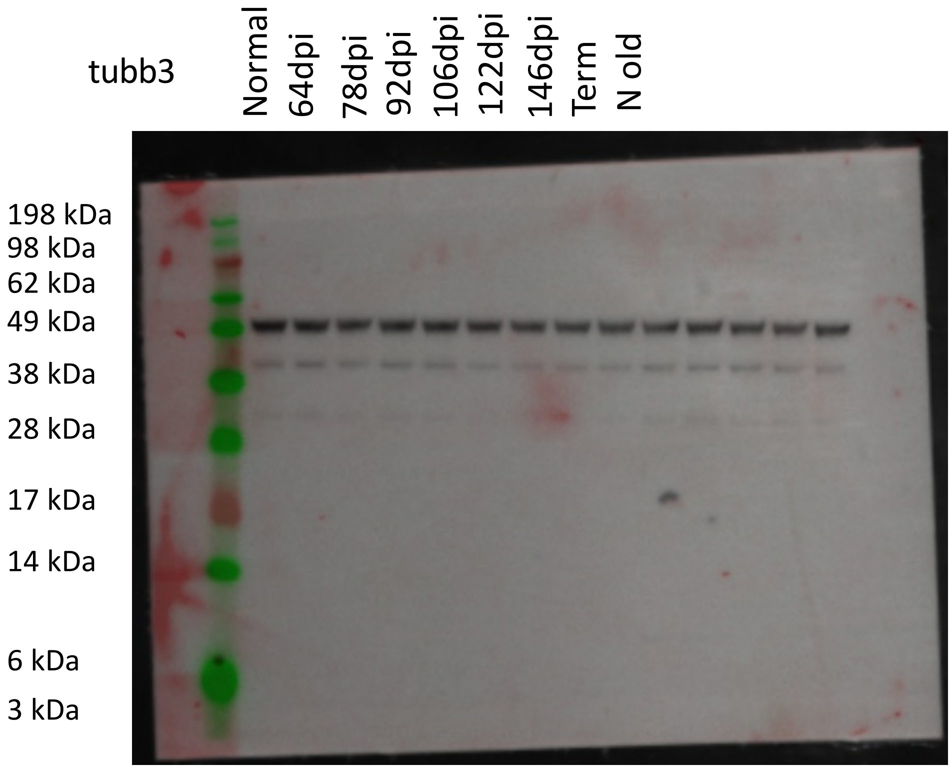
tubb3
Normal
64dpi
78dpi
92dpi
106dpi
122dpi
146dpi
Term
N old
198 kDa
98 kDa
62 kDa
49 kDa
38 kDa
28 kDa
17 kDa
14 kDa
6 kDa
3 kDa

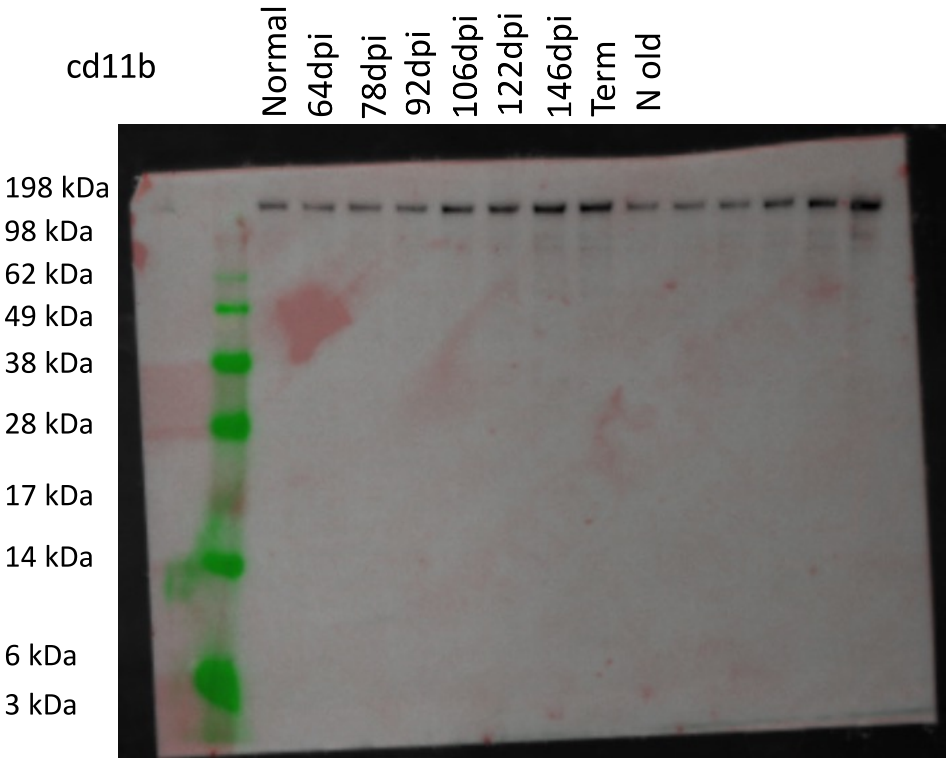
cd11b
Normal
64dpi
78dpi
92dpi
106dpi
122dpi
146dpi
Term
N old
198 kDa
98 kDa
62 kDa
49 kDa
38 kDa
28 kDa
17 kDa
14 kDa
6 kDa
3 kDa

Figure 9B

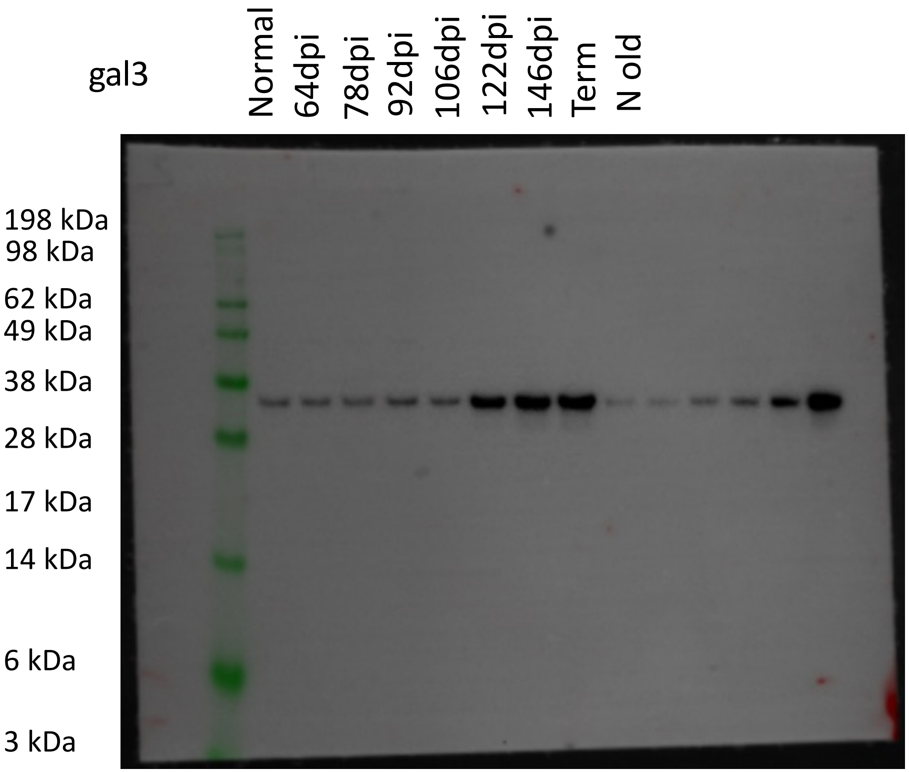
gal3
Normal
64dpi
78dpi
92dpi
106dpi
122dpi
146dpi
Term
N old
198 kDa
98 kDa
62 kDa
49 kDa
38 kDa
28 kDa
17 kDa
14 kDa
6 kDa
3 kDa

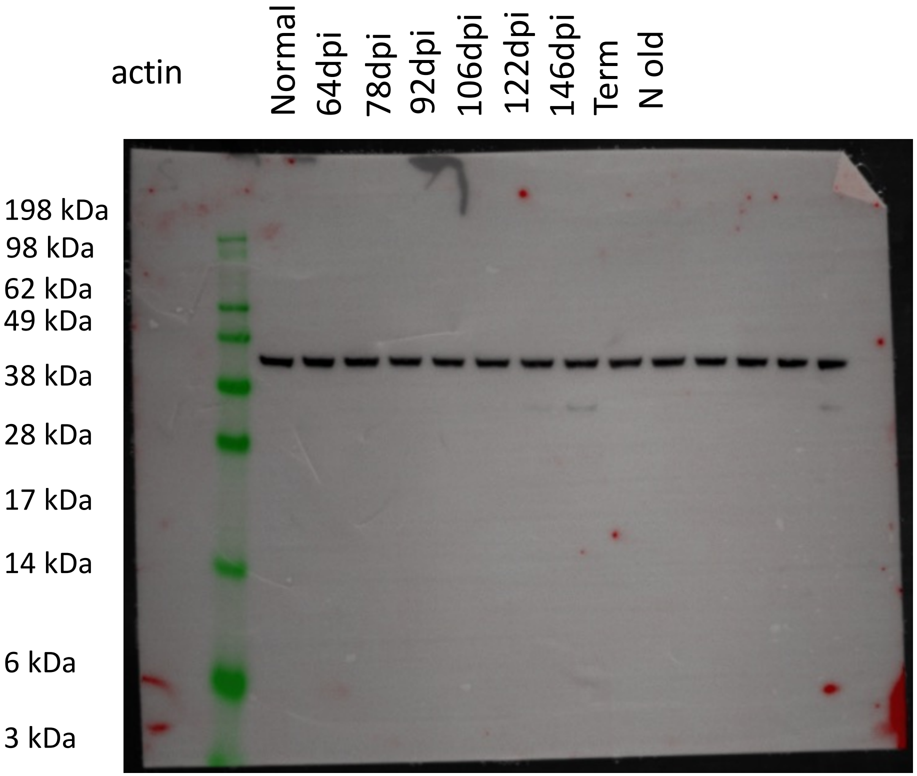
actin
Normal
64dpi
78dpi
92dpi
106dpi
122dpi
146dpi
Term
N old
198 kDa
98 kDa
62 kDa
49 kDa
38 kDa
28 kDa
17 kDa
14 kDa
6 kDa
3 kDa

Figure 11C

CD11b

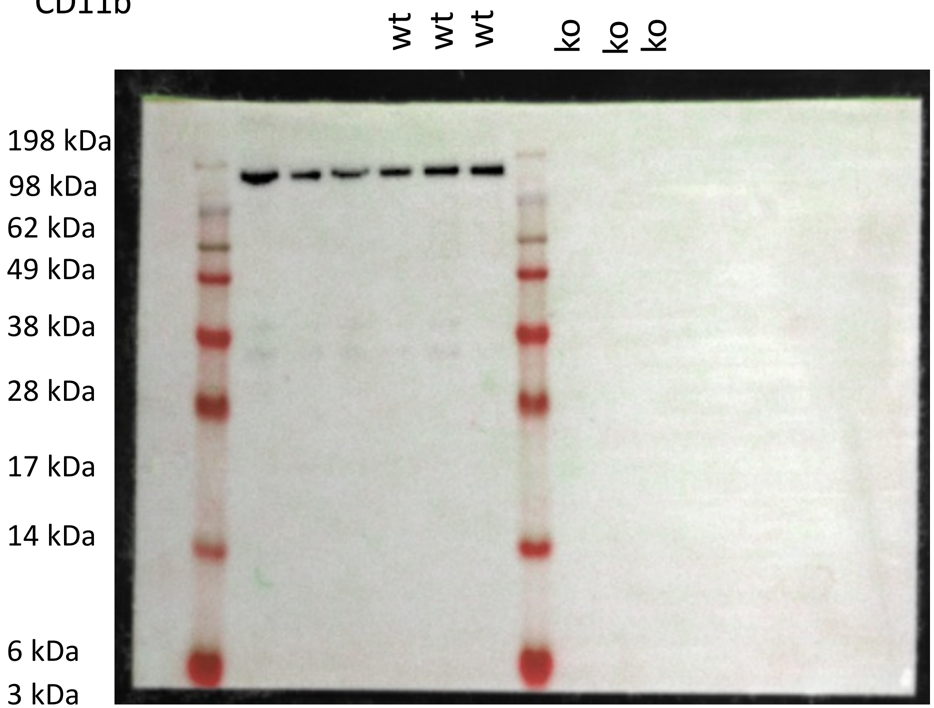

tubb3

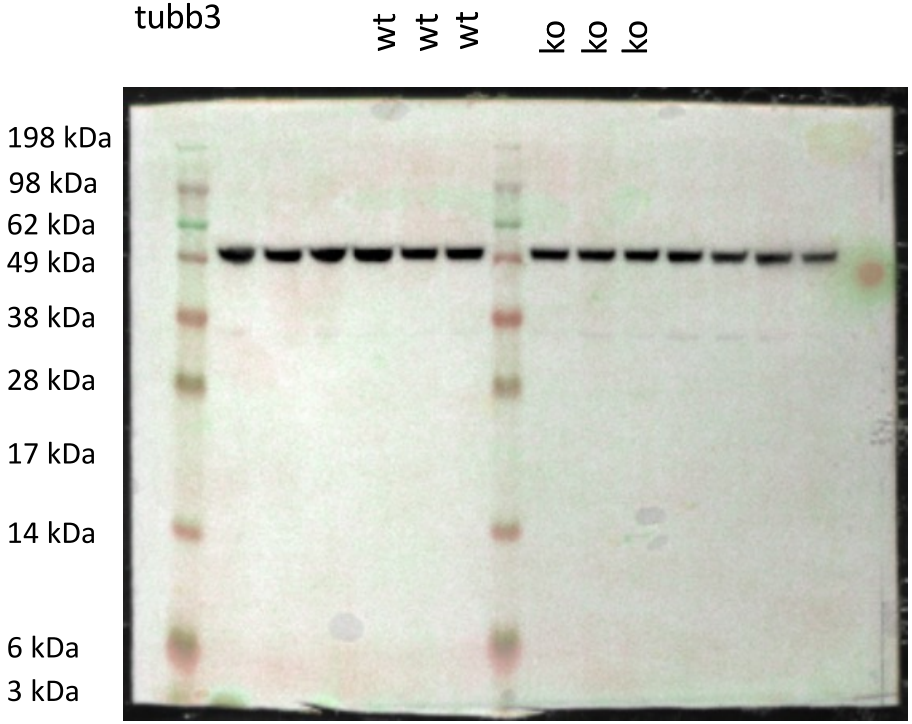

Figure 11C

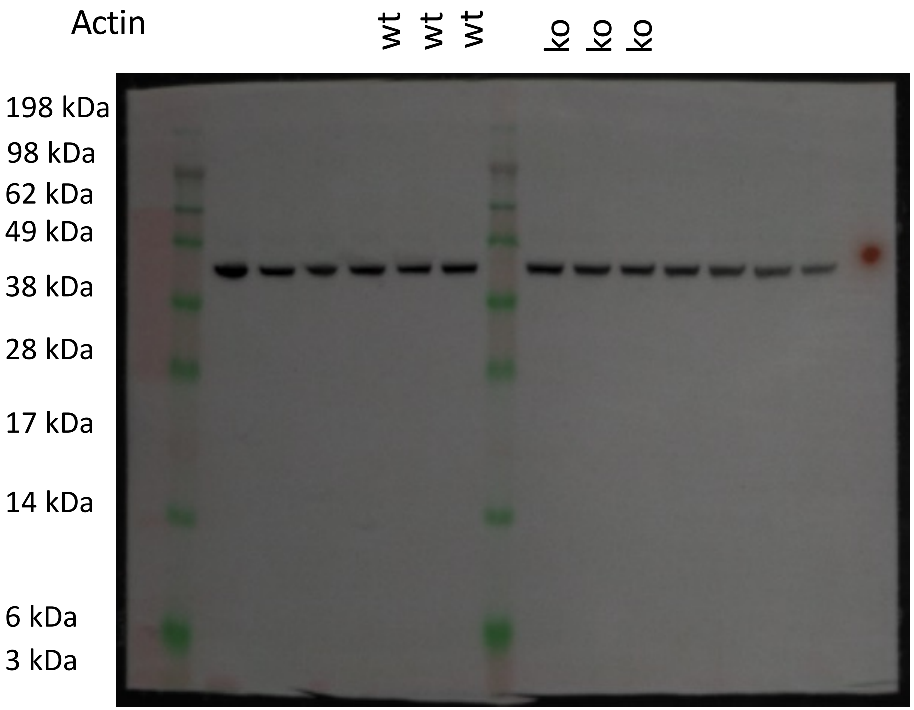
Actin
wt
wt
wt
ko
ko
ko
198 kDa
98 kDa
62 kDa
49 kDa
38 kDa
28 kDa
17 kDa
14 kDa
6 kDa
3 kDa

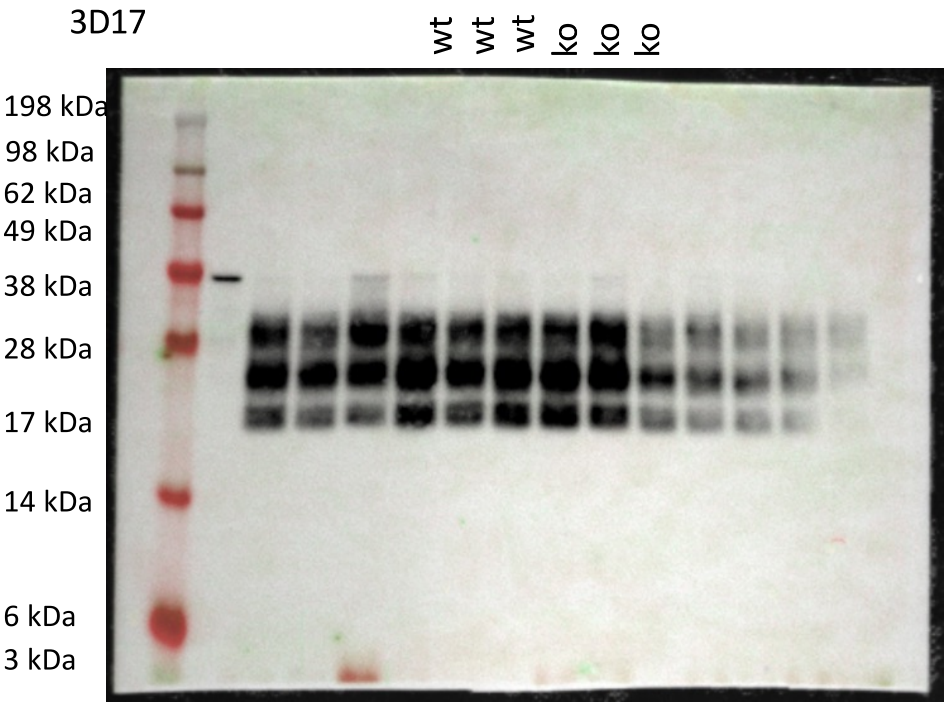
3D17
wt
wt
wt
ko
ko
ko
198 kDa
98 kDa
62 kDa
49 kDa
38 kDa
28 kDa
17 kDa
14 kDa
6 kDa
3 kDa

Figure 11C

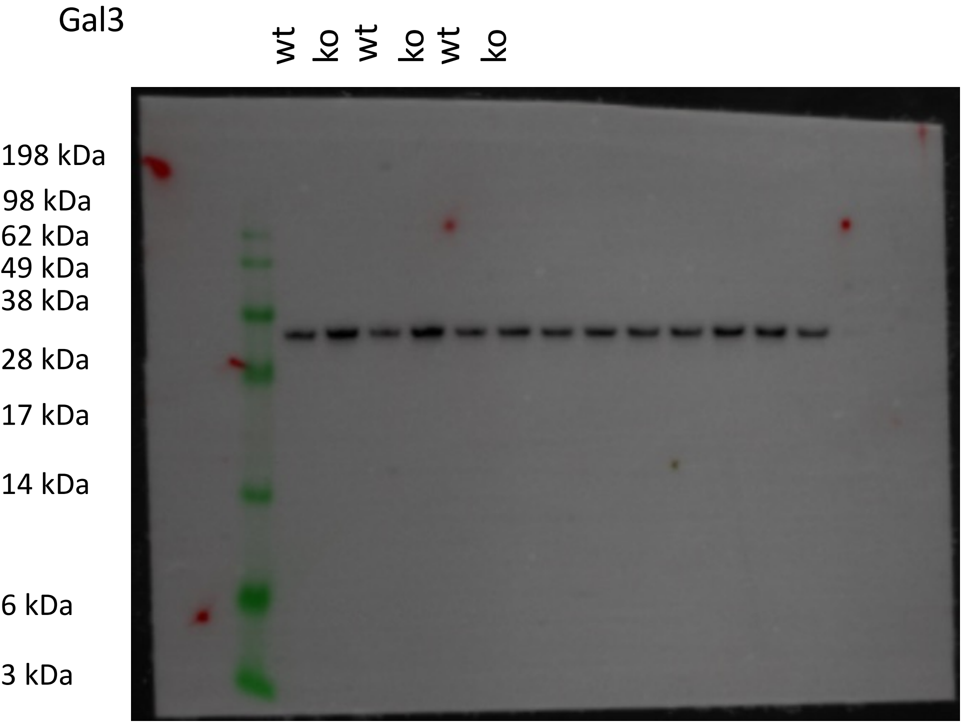
Gal3
wt
ko
wt
ko
wt
ko
198 kDa
98 kDa
62 kDa
49 kDa
38 kDa
28 kDa
17 kDa
14 kDa
6 kDa
3 kDa

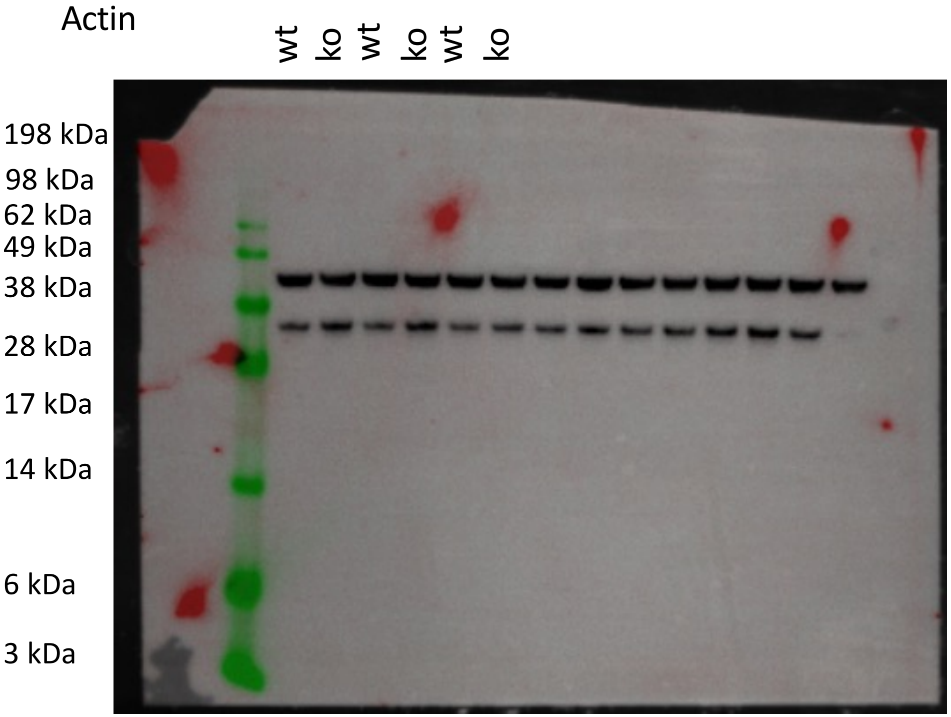
Actin
wt
ko
wt
ko
wt
ko
198 kDa
98 kDa
62 kDa
49 kDa
38 kDa
28 kDa
17 kDa
14 kDa
6 kDa
3 kDa
